# Short Cationic ACTH-Related Peptides Can Modulate the Na_V_1.8 Channel Functioning, Resulting in an Analgesic Effect

**DOI:** 10.3390/ijms27156792

**Published:** 2026-07-29

**Authors:** Ilya V. Rogachevskii, Arina D. Kalinina, Nadezhda A. Boichenko, Anna V. Berintseva, Iuliia V. Plakhova, Dmitriy M. Samosvat, Georgy G. Zegrya, Irina P. Butkevich, Viktor A. Mikhailenko, Valentina A. Penniyaynen, Svetlana A. Podzorova, Vladimir V. Kopat, Ilya V. Dukhovlinov, Boris V. Krylov

**Affiliations:** 1 Pavlov Institute of Physiology, Russian Academy of Sciences, 199034 Saint Petersburg, Russia; roggie_spb_ru@yahoo.com (I.V.R.);; 2 ATG Service Gene LLC., 199178 Saint Petersburg, Russia

**Keywords:** ACTH, short cationic peptides, Na_V_1.8 channel, patch-clamp method, organotypic tissue culture method, formalin test, conformational analysis, docking, nociception, analgesics

## Abstract

Full-length ACTH molecule and ACTH-related hexapeptide H-PKKRRP-OH are demonstrated by the patch-clamp method to decrease the Na_V_1.8 channel activation gating system effective charge in the nociceptive neuron membrane, while ACTH-related tetrapeptide Ac-KKRR-NH_2_ has no effect. ACTH(1–24), a fully functional ACTH mimetic, and H-PKKRRP-OH show analgesic effects in the formalin test in vivo. All peptides contain the cationic KKRR motif, but only H-PKKRRP-OH and ACTH(1–24) relieve acute pain, targeting the Na_V_1.8 channel as a receptor. This seemingly controversial result is explained by application of conformational analysis and blind docking. Though conformational analysis indicates that both H-PKKRRP-OH and Ac-KKRR-NH_2_ contain the cationic functional groups at the earlier suggested characteristic distance of 9–12 Å, Ac-KKRR-NH_2_ does not interact with the S4_I_ voltage sensor of the Na_V_1.8 channel activation gating system. The docking demonstrates that an extensive network of ligand–receptor ionic and hydrogen bonds involving D151, E157, R218, and R221 VSD_I_ residues, essential for the analgesic tripeptide Ac-KKK-NH_2_ binding, is formed upon the H-PKKRRP-OH binding. Particularly important are the ionic bonds between the H-PKKRRP-OH C-terminal carboxylate anion and the S4_I_ R218 and R221 guanidinium groups. The described mechanism of Na_V_1.8 channel modulation is fundamentally different from the effect of channel blockers.

## 1. Introduction

Adrenocorticotropic hormone (ACTH) is vital in managing stress. Stress is a universal response of a living organism to the action of adverse environmental factors or to homeostasis dysfunction [1]. The concept of stress was originally formulated by Hans Selye, who highlighted the fundamental role of endocrine mechanisms in adaptation to adverse stimuli [2]. According to this concept, pain should be considered not only as a sensory experience but also as a potent stressor capable of activating the nonspecific adaptive response of the human organism [3]. Within the framework of the general adaptation syndrome, activation of the hypothalamic–pituitary–adrenal (HPA) axis represents a central pathway linking stress to physiological regulation [4]. The release of glucocorticoids and related HPA hormones can modulate nociceptive processing at both peripheral and central levels [5]. Thus, pain and stress represent closely interconnected processes that integrate neuroendocrine and sensory mechanisms of adaptation. Cortisol, a steroid hormone synthesized in the adrenal cortex, is widely recognized as the primary stress hormone [6,7]. Its secretion is stimulated by ACTH produced by the pituitary gland, while the release of ACTH itself is regulated by the hypothalamic corticotropin-releasing hormone [8]. This ensures neuroendocrine modulation of metabolic and immune reactions in response to stressors through the negative feedback mechanism. An acute stress reaction is essential for rapid body adaptation, prompt response, and subsequent homeostasis recovery. Alas, stress becomes chronic if the stressor is not eliminated in due time. In such cases, the negative feedback mechanism usually malfunctions, resulting in permanently elevated levels of the HPA axis hormones, which can eventually lead to organ and tissue damage, as well as the development of cardiovascular diseases, anxiety–depressive disorders, and immune system dysfunctions [1].

A similar correlation is observed in the nociceptive system functioning [9]. Undoubtedly, pain is evolutionarily necessary for the body’s survival and protection. However, persistent and prolonged pain sensations that become chronic do not carry much vitally essential information and become a burden [10]. Chronic pain leads to specific nervous system disorders, which distinguishes it from pain as a symptom. Therefore, chronic pain should be regarded as a distinct neuropathological syndrome that requires targeted treatment [11]. Although acute stress produces analgesia due to the activation of endogenous opioidergic and catecholaminergic systems [12,13], chronic stress has the opposite effect, leading to pain sensitization. Glucocorticoids such as cortisol, which normally suppress inflammatory processes, become less effective with prolonged hypersecretion and contribute to neurotoxicity [14,15]. Chronic stress, characterized by persistent reactivation of the HPA axis and elevated hormone levels, may potentially exacerbate pathological nociceptive conditions [16]. Neuroimaging and behavioral data indicate that the corticolimbic system is engaged not only in the regulation of the affective and motivational aspects of pain but also in the regulation of the HPA axis via feedback mechanisms predominantly mediated by glucocorticoid receptors of the medial prefrontal cortex and hippocampus. Chronic pain and long-term stress lead to persistent neuroplastic changes in the corticolimbic system structures, exemplified by reduced hippocampal volume, loss of dendritic spines in the prefrontal cortex, and elevated amygdala excitability [17]. These alterations disrupt the homeostasis of the HPA axis and contribute to the development of a maladaptive response, which maintains high basal cortisol secretion [18]. Thus, HPA axis hormones not only regulate the organismal response to stress but also directly participate in the modulation of pain processing at both spinal and supraspinal levels of the nociceptive system.

The role of glucocorticoids, particularly cortisol, in pain modulation and stress-related alterations in nociception is well established [19,20]. Dysregulation of cortisol signaling under chronic stress conditions has been shown to contribute to increased pain sensitivity and the development of hyperalgesia. In contrast, the involvement of ACTH in nociceptive modulation remains insufficiently characterized. Although ACTH release is tightly coupled to nociceptive input and can be directly induced by painful stimuli, as demonstrated in experimental models such as the formalin test [21], its direct role in the regulation of nociceptive signaling pathways has been far less extensively studied. The only example is the results of experiments in vivo on anesthetized animals: the pain threshold was measured as the electric current value at which rats displayed a tail withdrawal response to electrical stimulation [22]. ACTH-induced analgesia had been completely eliminated by the suppression of glucocorticoid production together with the blockade of opioid receptors. The analgesic effect of ACTH was shown to be mediated by two different mechanisms: (1) a rapidly acting (from 3 to 15 min) mechanism associated with opioid receptors and not related to glucocorticoids, and (2) a delayed (from 15 to 30 min) mechanism associated with glucocorticoids but not opioid receptors.

**Figure 1:**
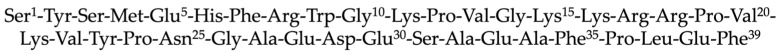
Amino acid sequence of human ACTH. The residues 1–24 are conserved within the mammalian species.

The present study examines the direct effect of ACTH and several ACTH-related peptides on the slow Na_V_1.8 sodium channel, the key molecular target of the peripheral nociceptive system [23]. ACTH is an endogenous peptide hormone with structural features potentially compatible with direct interactions with ion channel targets, and it is also of special interest because the ACTH molecule includes a cationic motif of two lysine (K) and two arginine (R) residues (Figure 1) [24]. We have previously demonstrated that several short lysine- and arginine-containing peptides (up to six amino acid residues) modulate the Na_V_1.8 channel functional activity, resulting in the pronounced antinociceptive effect at the organismal level confirmed in vivo using the formalin test [25,26,27,28]. It has been shown that the short attacking peptide should be active when it has at least three cationic side chain functional groups, either arginine guanidinium groups or lysine amino groups, forming a triangle of positive charges with the side lengths of 9–12 Å.

ACTH natively contains the K^15^KRR^18^ motif of four mixed cationic residues. In accordance with our model, it is this sequence that should be responsible for the direct ligand–receptor ACTH binding with the Na_V_1.8 channel. Other cationic ACTH residues (R^8^, K^11^, K^21^) do not form a cluster and appear to be rather distant from the KKRR sequence. It is also worth noting that the KKRR sequence is followed by a proline (P^19^). Due to its conformational rigidity, this residue kinks the peptide backbone and likely plays a particular role in stabilizing the low-energy structures of short cationic peptides [26]. Another proline (P^12^) is found two residues prior to the KKRR sequence.

To test the idea that a cationic ACTH-related fragment might modulate the Na_V_1.8 channel functional activity, we have custom designed and synthesized two short peptides, Ac-KKRR-NH_2_ and H-PKKRRP-OH. The tetrapeptide Ac-KKRR-NH_2_ is a native ACTH fragment, N-terminally acetylated and C-terminally amidated in order to protect it against the action of proteolytic endopeptidases during delivery to its molecular target in vivo. The hexapeptide H-PKKRRP-OH includes a native KKRRP sequence preceded by a proline, and it should be rather resistant to proteolytic decomposition due to the presence of proline residues at both ends of the molecule.

Our research methodology [25,26,27,28] is based on the patch-clamp investigation of the effects of short cationic peptides on the Na_V_1.8 channel functional activity in living neurons, which makes it possible to predict the analgesic effect of the attacking molecules at the organismal level. The organotypic tissue culture method is applied to find out whether the studied molecules affect nerve tissue growth. Theoretical conformational analysis and further blind docking with the Na_V_1.8 channel molecule elucidate the ligand–receptor binding mechanism at the atomic level, thus refining the experimental predictions. Finally, the formalin test is used to verify the analgesic effect in vivo.

Regrettably, ACTH is a long, linear, and relatively loosely structured polypeptide comprised of 39 amino acid residues. Therefore, theoretical analysis is not expected to provide much reliable data regarding its physiologically relevant conformations upon binding with the Na_V_1.8 channel, which also makes ACTH docking not very informative. Considering the above, the full set of experimental and calculational methods has been applied only to the two short ACTH-related peptides, Ac-KKRR-NH_2_ and H-PKKRRP-OH.

## 2. Results

### 2.1. The Patch-Clamp Method

**Figure 2:**
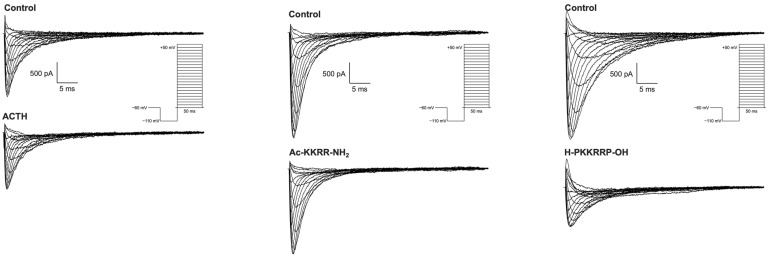
Families of Na_V_1.8 currents in the control experiments (**above**) and after extracellular application of ACTH- and ACTH-related cationic peptides at 100 nM (**below**). Leakage and capacitive currents were subtracted automatically.

**Figure 3:**
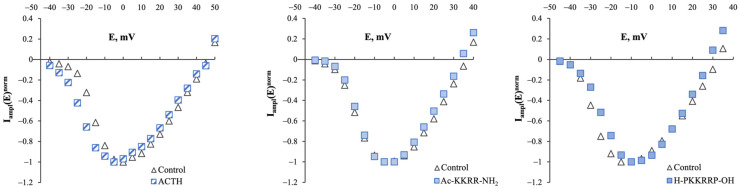
Normalized Na_V_1.8 channel amplitude current–voltage functions I_ampl_(E) in the control experiments and after extracellular application of ACTH- and ACTH-related cationic peptides at 100 nM.

**Figure 4:**
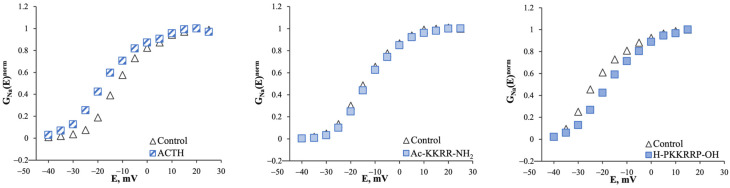
Normalized voltage sensitivity of the Na_V_1.8 channel chord conductance G_Na_(E)^norm^ in the control experiments and after extracellular application of ACTH- and ACTH-related cationic peptides at 100 nM.

**Figure 5:**
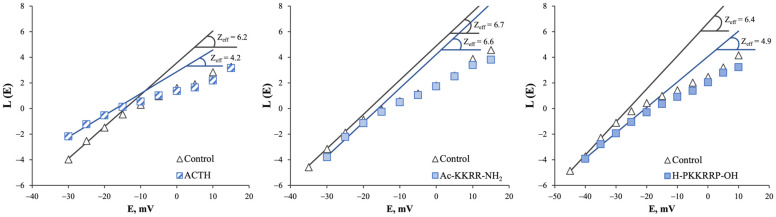
Evaluation of Z_eff_, the effective charge of the Na_V_1.8 activation gating system, in the control experiments and after extracellular application of ACTH- and ACTH-related cationic peptides at 100 nM.

The families of Na_V_1.8 sodium currents were recorded under control conditions and after extracellular application of either ACTH, Ac-KKRR-NH_2_, or H-PKKRRP-OH at 100 nM (Figure 2). Subsequently, the amplitude current–voltage functions I_ampl_(E) were constructed. When observed, the changes in the steepness of the left I_ampl_(E) branch were used to assess how the Na_V_1.8 activation gating system voltage sensitivity had been modulated in response to the peptide application (Figure 3). These changes were visualized more clearly using G_Na_(E), the normalized voltage dependencies of the chord conductance. The steepness of the initial S-shaped G_Na_(E) fragment reflects the characteristic features of the voltage sensitivity of the Na_V_1.8 channel activation process (Figure 4). The ligand–receptor binding of ACTH and H-PKKRRP-OH to the Na_V_1.8 channel resulted in a change in the Z_eff_ values, which were determined by constructing the logarithmic voltage sensitivity function L(E) in each experiment (Figure 5).

**Figure 6:**
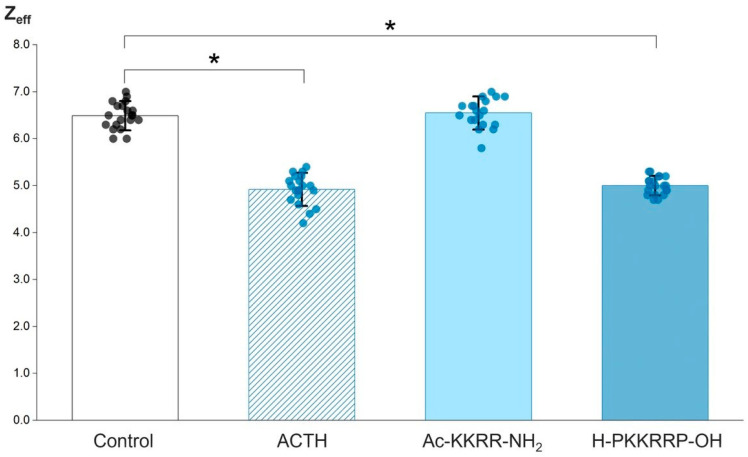
Values of Z_eff_, the effective charge of the Na_V_1.8 channel activation gating system, in the control experiments and after extracellular application of ACTH- and ACTH-related cationic peptides at 100 nM. Data are presented as mean ± SEM. Statistically significant differences between the control and experimental values are designated with the asterisks (* *p* < 0.05).

Across the experiments, a statistically significant decrease in Z_eff_ as compared with the control (6.5 ± 0.3; *n* = 20) was observed following the application of ACTH (4.9 ± 0.3; *n* = 20) and H-PKKRRP-OH (5.0 ± 0.2; *n* = 20) at 100 nM. In contrast, the Z_eff_ value obtained after the Ac-KKRR-NH_2_ application at 100 nM (6.6 ± 0.2; *n* = 20) did not differ from the control (Figure 6).

### 2.2. Organotypic Tissue Culture Method

**Figure 7:**
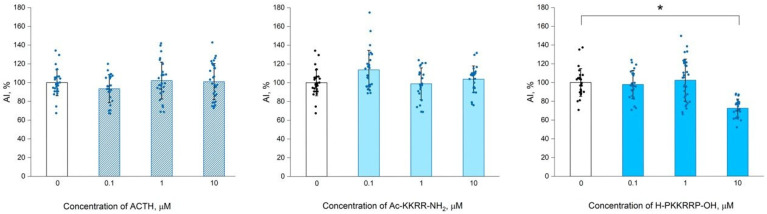
Effects of ACTH- and ACTH-related cationic peptides on neurite growth in DRG sensory neuron explants (third day of culturing). The ordinate axis—area index (AI, %). Zero concentration of the tested substances indicates the control explants. Data are presented as mean ± SEM. Statistically significant differences between the control and experimental values are designated with the asterisks (* *p* < 0.05).

Introduction of either ACTH or Ac-KKRR-NH_2_ into the culturing medium at the concentrations of 0.1 μM (ACTH, *n* = 25; Ac-KKRR-NH_2_, *n* = 27), 1 μM (ACTH, *n* = 29; Ac-KKRR-NH_2_, *n* = 23), and 10 μM (ACTH, *n* = 30; Ac-KKRR-NH_2_, *n* = 25) did not significantly affect the DRG neurite growth (Figure 7). The AI of the experimental explants did not differ from the control value (*n* = 29).

Unlike ACTH and Ac-KKRR-NH_2_, H-PKKRRP-OH was demonstrated to have a neurite-inhibiting effect, indicating the putative ability of this peptide to activate the membrane metabotropic receptors that trigger the intracellular signaling cascades controlling the neurite growth. At 10 μM, the peptide significantly inhibited the DRG neurite growth (Figure 7). The AI of the experimental explants was 27 ± 5% (*n* = 29) lower than the control value (*n* = 27). At lower concentrations, 0.1 μM (*n* = 25) and 1 μM (*n* = 32), H-PKKRRP-OH did not affect the neurite growth.

### 2.3. Conformational Analysis

**Figure 8:**
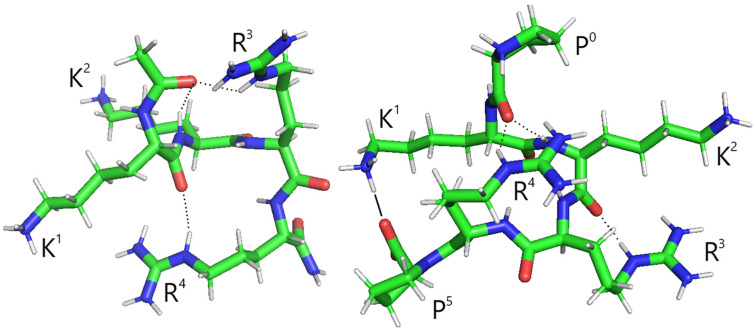
Lowest-energy conformations of Ac-KKRR-NH_2_ (**left**) and H-PKKRRP-OH (**right**) at dielectric constant ε = 10 with the residue numbering. Carbon, green; oxygen, red; nitrogen, blue; hydrogen, white. Hydrogen bonds are indicated with dotted lines; the ionic bond between the K^1^ side chain amino group and the C-terminal carboxylic group is shown with a solid line.

Due to the presence of the lysine and arginine positively charged side chain functional groups, Ac-KKRR-NH_2_ and H-PKKRRP-OH bear a total charge of +4. Their lowest-energy conformations obtained at the dielectric constant value ε = 10 are shown in Figure 8. The terminal Ac-KKRR-NH_2_ functional groups were protected by N-acetylation and C-amidation. The H-PKKRRP-OH molecule was considered in the zwitter-ion form; thus, the P^0^ N-terminal secondary amino group was charged positively, while the P^5^ C-terminal carboxylic group was charged negatively. To keep consistent numbering, the first proline residue in H-PKKRRP-OH is named P^0^. It should also be specified that, from now on, the residue numbers of the ACTH-related peptides will be shown in superscripts to distinguish them from the Na_V_1.8 channel residue numbers.

The lowest-energy conformations of both studied ACTH-related short cationic peptides contain three intramolecular hydrogen bonds. Two of them attach the R^3^ and R^4^ guanidinium groups to the backbone carbonyls, while another one stabilizes the backbone conformation between P^0^ (or acetyl N-capping group in the case of Ac-KKRR-NH_2_) and R^2^. In H-PKKRRP-OH, the C-terminal carboxylate anion forms a salt bridge with the K^1^ side chain amino group. The values of distances between the positively charged side chain functional groups in Ac-KKRR-NH_2_ and H-PKKRRP-OH averaged over the entire conformational ensemble and over a number of smaller low-energy subensembles are given in Table 1. The results obtained at ε = 10 and ε = 80 are very similar, so only the data related to ε = 10 are presented and discussed further.

As suggested earlier [25,26,27,28], a subensemble that realistically describes the low-energy conformational space of short cationic peptides should contain 1–2% of the total number of generated conformations. Therefore, $\mu_{ij},$ the distances between the positively charged side chain functional groups in the low-energy conformational space of the studied peptides are obtained at the energy cutoff of 5 kcal/mol for Ac-KKRR-NH_2_ and of 6 kcal/mol for H-PKKRRP-OH (Table 1).

In the Ac-KKRR-NH_2_ molecule, the K^1^–K^2^, K^1^–R^3^, and K^2^–R^3^ distances remain fairly constant over all selected subsensembles, while the K^1^–R^4^, K^2^–R^4^, and R^3^–R^4^ distances noticeably decrease with the decrease in the energy cutoff. The observed trend indicates that the Ac-KKRR-NH_2_ low-energy conformational space is characterized by the formation of a hydrogen bond between the R^4^ guanidinium group and the K^1^ backbone carbonyl oxygen (Figure 8). Rather counterintuitively, this hydrogen bond brings the R^4^ guanidinium moiety closer to other cationic side chain functional groups of the studied peptide.

In the H-PKKRRP-OH low-energy conformational space, the K^1^–K^2^ and K^1^–R^3^ distances are longer, and the K^1^–R^4^ distance is shorter than the corresponding distances averaged over the entire conformational space. This effect is due to the formation of an ionic bond between the K^1^ side chain amino group and the C-terminal carboxylate anion. Also, the formation of a hydrogen bond between the R^3^ guanidinium group and the K^2^ backbone carbonyl oxygen decreases the K^2^–R^3^ distance and increases the R^3^–R^4^ distance.

### 2.4. Peptide Docking with the Na_V_1.8 Channel Molecule

**Figure 9:**
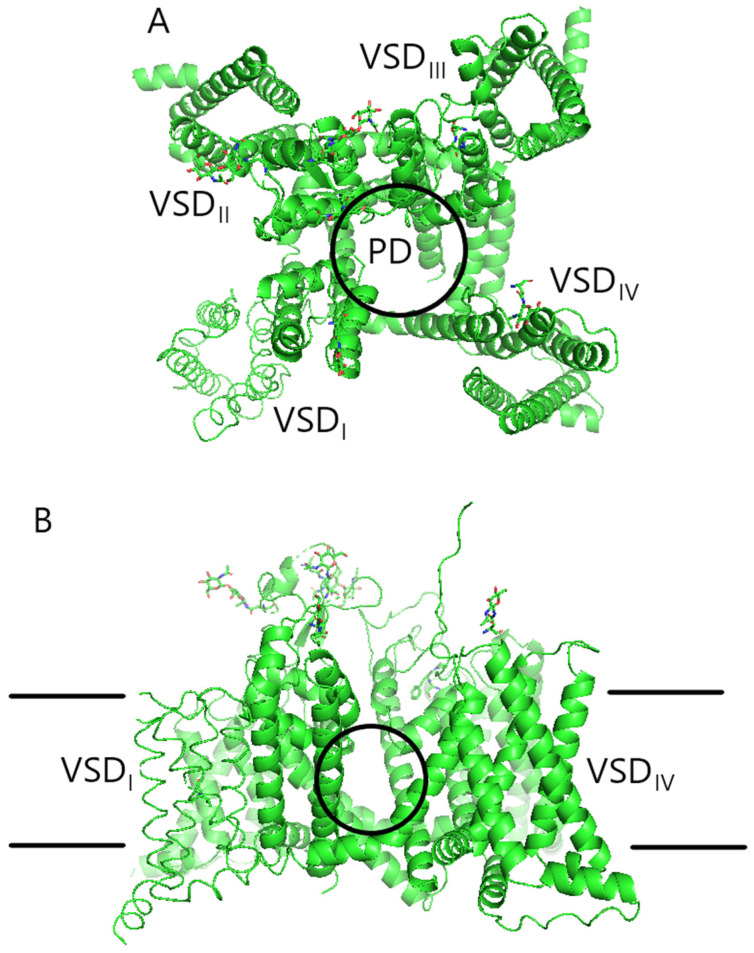
Extracellular (**A**) and lateral (**B**) views of the Na_V_1.8 channel molecule. The voltage-sensing domains VSD_I_–VSD_IV_ are attached to the pore domain PD in a clockwise manner. The cation-permeating pore and the lateral fenestration are highlighted with black circles. The approximate position of the neuron membrane presented with solid black lines is taken from [29].

The Na_V_1.8 channel molecule comprises four nonidentical domains D_I_–D_IV_, any of which is built of six S1–S6 transmembrane segments. The S5 and S6 segments contribute to the pore domain (PD) that contains the Na^+^ cation permeation pathway with the hydrophobic central cavity in the middle and lateral fenestrations opening from the central cavity into the membrane lipid bilayer. The S1–S4 segments of D_I_–D_IV_ constitute the corresponding voltage-sensing domains (VSD_I_–VSD_IV_) that control the Na_V_1.8 channel voltage sensitivity. The S4 segments are considered the voltage sensors, as they contain a number of cationic residues (from 4 to 6) that move up and down within the neuron membrane during the channel working cycle. Two projections of the Na_V_1.8 channel molecule, extracellular and lateral, are presented in Figure 9.

**Figure 10:**
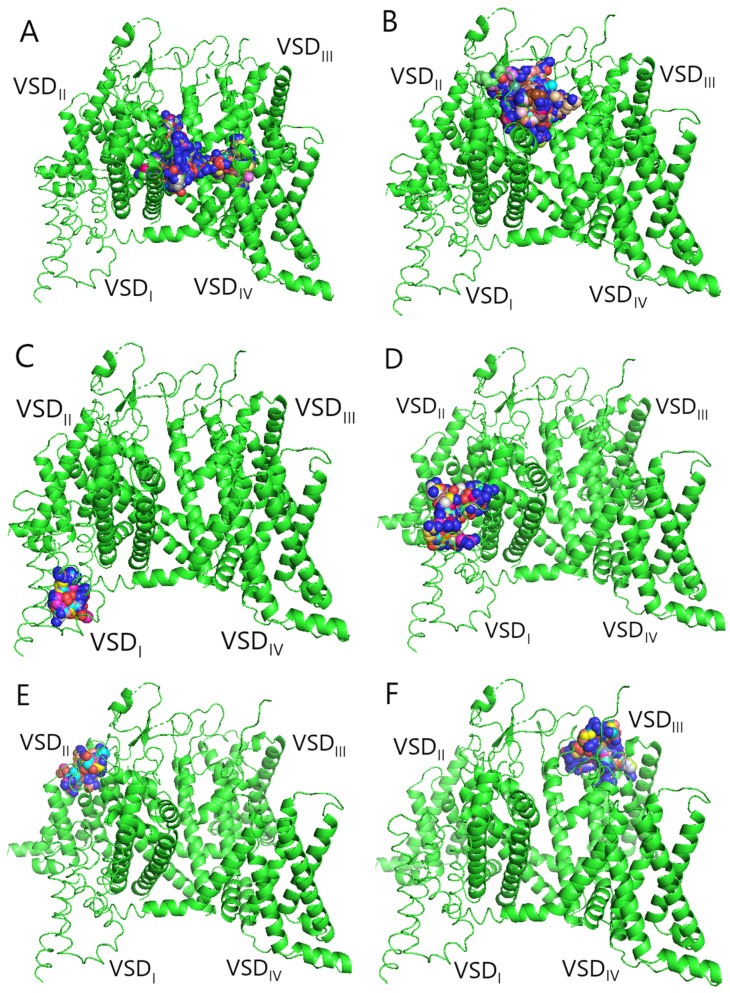
Ac-KKRR-NH_2_ binding sites (**A**–**F**) located by its blind docking with the Na_V_1.8 channel molecule. The Na_V_1.8 channel is shown as a green cartoon. Superimpositions of all conformations docked to the corresponding sites are shown as colored spheres. Hydrogen atoms are not presented for clarity.

**Figure 11:**
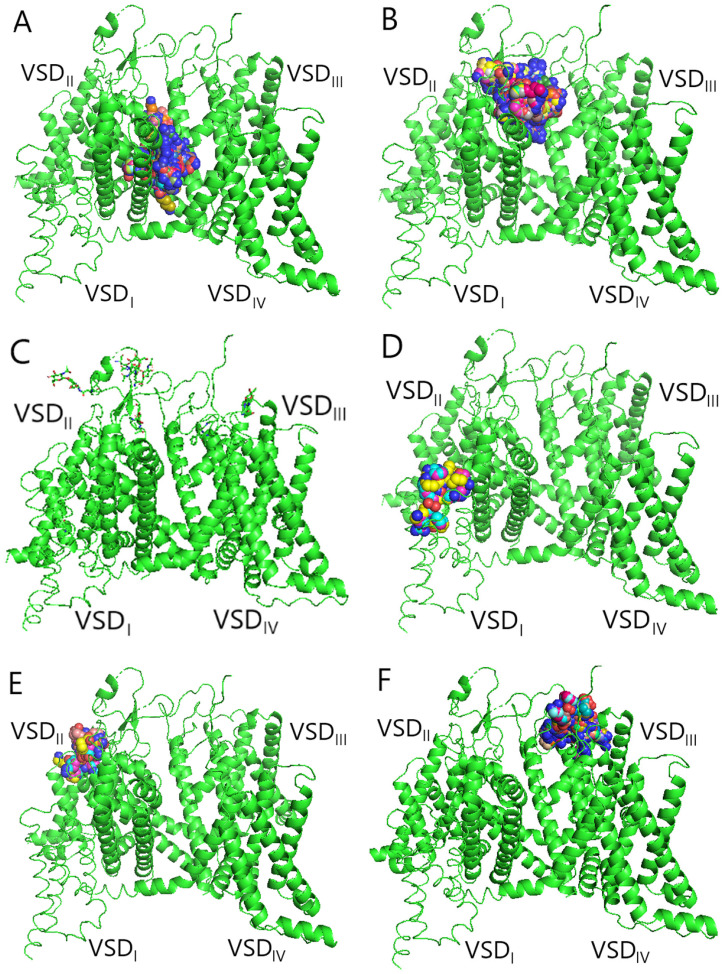
H-PKKRRP-OH binding sites (**A**–**F**) located by its blind docking with the Na_V_1.8 channel molecule. The Na_V_1.8 channel is shown as a green cartoon. Superimpositions of all conformations docked to the corresponding sites are shown as colored spheres. Hydrogen atoms are not presented for clarity. Subfigure (**C**) displays solely the lateral projection of the Na_V_1.8 channel molecule, as H-PKKRRP-OH does not bind to the site C.

The applied energy and RMSD selection criteria allowed the isolation of 75 unique low-energy Ac-KKRR-NH_2_ conformations and 51 H-PKKRRP-OH conformations. This corresponds to 300 and 204 single docking runs for Ac-KKRR-NH_2_ and H-PKKRRP-OH, respectively, all of which have been successful. Six clusters of docked conformations (sites A–F) were identified for Ac-KKRR-NH_2_ (Figure 10), and only five for H-PKKRRP-OH (Figure 11). The parameters of Ac-KKRR-NH_2_ and H-PKKRRP-OH conformational ensembles docked at the identified binding sites in the Na_V_1.8 channel molecule are presented in Table 2.

Though sites A and B account for a large fraction of the obtained docking poses, sites A–C were excluded from further discussion after inspection of the results. The blind docking procedure does not take into consideration the fact that the attacking peptides would have to penetrate through the neuronal membrane or the cation-permeating pore to dock at the central cavity or at the Na_V_1.8 channel intracellular side. Also unconsidered is the fact that the peptide binding does not block the Na_V_1.8 currents, which makes their docking within the pore also highly improbable.

Site A includes the selectivity filter and the hydrophobic central cavity together with the II–III and III–IV fenestrations, the H-PKKRRP-OH molecule being too large to dock into the III–IV fenestration. Site B is located at the pore entrance within the extracellular loop (ECL) region. Binding to these areas is characteristic of the channel blockers [30], which is not the case for the investigated peptides, as their extracellular application does not inhibit the Na_V_1.8 currents in our patch-clamp experiments. Moreover, binding to site A would require the ligand to pass through the selectivity filter into the central cavity, which does not seem probable for the relatively bulky H-PKKRRP-OH molecule. Site C is positioned on the VSD_I_ intracellular side, and it is also irrelevant for further consideration simply because the active H-PKKRRP-OH molecule does not bind to this site. In addition, transmembrane penetration of the attacking peptide would be required to reach site C. This is why the retained VSD-associated poses (sites D–F) are considered more mechanistically relevant than the more frequently sampled sites.

Site D is located on the VSD_I_ extracellular side. The studied peptides bind in the hydrophilic cleft between the S1_I_/S2_I_ and S3_I_/S4_I_ segments. Importantly, the side chains of cationic residues R215, R218, R221, and K224 belonging to the S4_I_ segment and forming the voltage sensor are oriented toward this cleft and thus expected to interact with the docked peptides. In the native environment, these residues are stabilized by a number of intramolecular interactions: R215 forms ionic bonds with the aspartate D151 and glutamate E157, and a hydrogen bond with the glutamine Q807; R218 and R221 form hydrogen bonds with the threonine T161 and asparagine N143, correspondingly [31].

**Figure 12:**
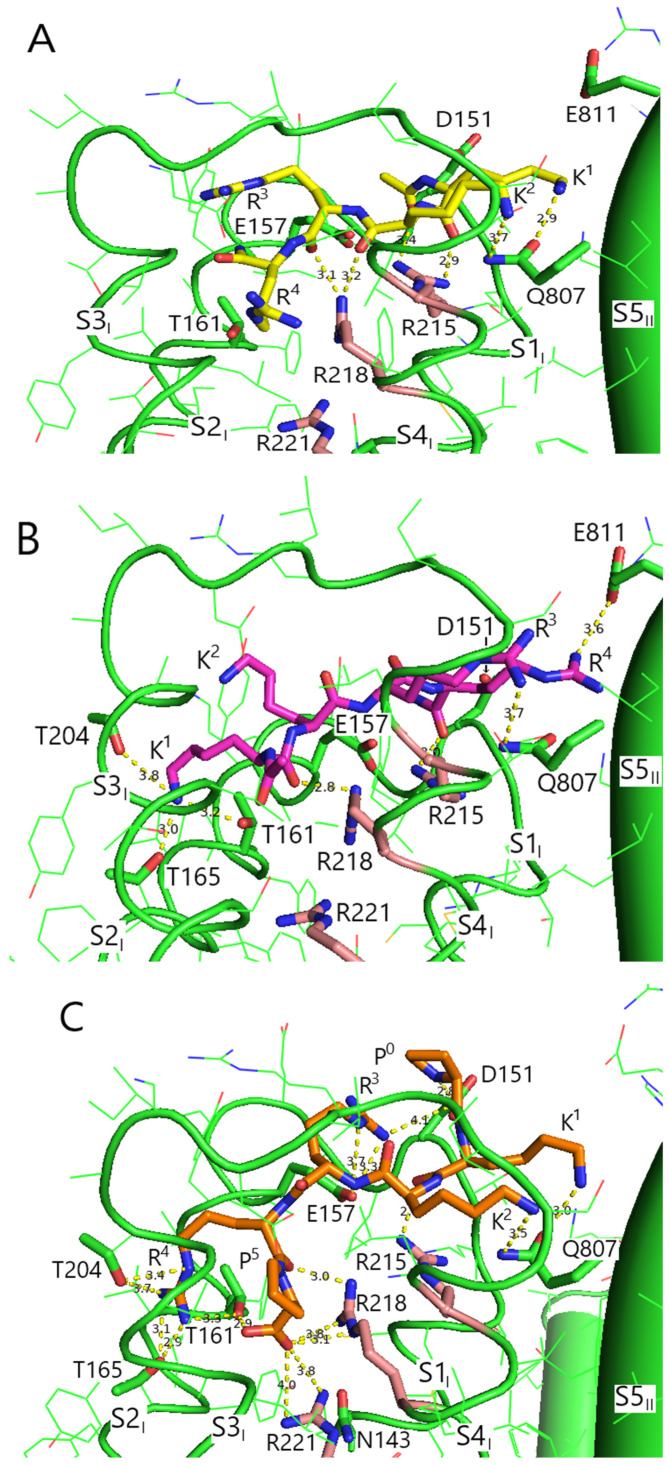
Lateral views of the lowest-energy Ac-KKRR-NH2 (**A**,**B**) and H-PKKRRP-OH (**C**) docking poses with site D. The Na_V_1.8 channel is shown as a green cartoon with lines. Cationic residues of the S4_I_ voltage sensor are displayed with pink sticks. Several amino acid residues important for the peptide binding are highlighted with green sticks. Docked peptides are shown in colored sticks. Standard atom coloring is used: oxygen, red; nitrogen, blue. Hydrogen atoms are not presented for clarity. Intermolecular ionic and hydrogen bonds are displayed with yellow dashed lines. Distances between heavy atoms in a bond are given in angstroms.

The lowest-energy docking poses of the attacking peptides at site D are shown in Figure 12. The Ac-KKRR-NH_2_ molecule is found to bind in two different orientations, which were detected almost equally frequently (7 and 6 conformations, respectively). When the N-end of the molecule is positioned closer to the PD S5_II_ segment (E = −7.1 kcal/mol) (Figure 12A), the ligand–receptor complex is stabilized with a number of hydrogen bonds: the K^1^ and K^2^ amino groups interact with the Q807 carboxamide group on the S5_II_ segment; the N-terminal acetyl and K^1^ backbone carbonyl oxygens with the R215 guanidinium group; the K^2^ and R^3^ backbone carbonyl oxygens with the R218 guanidinium group. When the C-end of the Ac-KKRR-NH_2_ molecule is located closer to the pore (E = −7.1 kcal/mol) (Figure 12B), the K^1^ amino group is hydrogen bonded to the T161, T165, and T204 hydroxyls; the R^3^ guanidinium group to the Q807 carboxamide; the K^1^ backbone carbonyl oxygen to the R218 guanidinium group; and the R^4^ backbone carbonyl oxygen to the R215 guanidinium group. Finally, R^4^ forms a salt bridge with E811, which, like Q807, belongs to the PD S5_II_ segment.

It is worth noting that the formation of an intermolecular ionic bond between one of the Ac-KKRR-NH_2_ positively charged groups and D151 is detected only in 5 cases of the peptide docking at site D out of 13. Moreover, ionic bonds between the attacking peptide and E157 are not detected at all. Thus, the strongest intermolecular interactions of the Ac-KKRR-NH_2_ positively charged functional groups are observed with Q807 and E811 PD residues. The tetrapeptide binding to site D barely affects the S4_I_ voltage sensor cationic residues and their native environment, with the exception of several hydrogen bonds formed between the Ac-KKRR-NH_2_ backbone carbonyl oxygens and the R215 and R218 guanidinium groups. In general, the Ac-KKRR-NH_2_ molecule interacts with VSD_I_ rather weakly.

The N-end of the H-PKKRRP-OH molecule (E = −7.7 kcal/mol) (Figure 12C), upon binding to site E, is located closer to the S5_II_ segment. A number of intermolecular ionic bonds are found: between the P^0^ N-terminal secondary amino group and the D151 carboxylate anion; between the R^3^ guanidinium group and the D151 and E157 carboxylate anions; and, most importantly, between the P^5^ C-terminal carboxylate anion and the R218 and R221 guanidinium groups. These interactions are not detected upon the Ac-KKRR-NH_2_ binding to this site. In addition to ionic bonds, the H-PKKRRP-OH molecule also forms several intermolecular hydrogen bonds: the K^1^ and K^2^ amino groups interact with the Q807 carboxamide; the R^4^ guanidinium group with the T161, T165, and T204 hydroxyls; the P^5^ C-terminal carboxylate anion with the T161 hydroxyl. Two more hydrogen bonds involve the H-PKKRRP-OH backbone atoms: the K^1^ carbonyl oxygen interacts with the R215 guanidinium group, and the R^4^ carbonyl oxygen interacts with the R218 guanidinium group.

Site E is situated on the VSD_II_ extracellular side. Similar to what has been observed upon their binding to site D, the peptides dock in the hydrophilic cleft between the S1_II_/S2_II_ and S3_II_/S4_II_ segments. The docking results indicate that only one of the five S4_II_ voltage sensor cationic residues (R756, R759, R762, K765, or K768) can directly interact with the attacking peptides, the closest to the extracellular side of the neuron membrane arginine R756. The R756 side chain conformation is stabilized by several intramolecular interactions of the guanidinium group: an ionic bond with the glutamate E685 anion, as well as hydrogen bonds with the serine S753 hydroxyl and the asparagine N1301 carboxamide.

**Figure 13:**
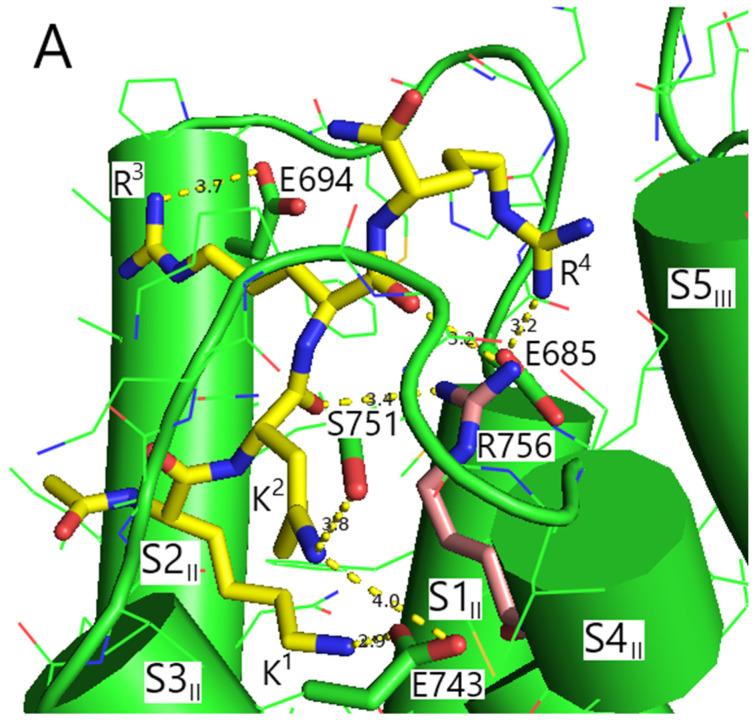

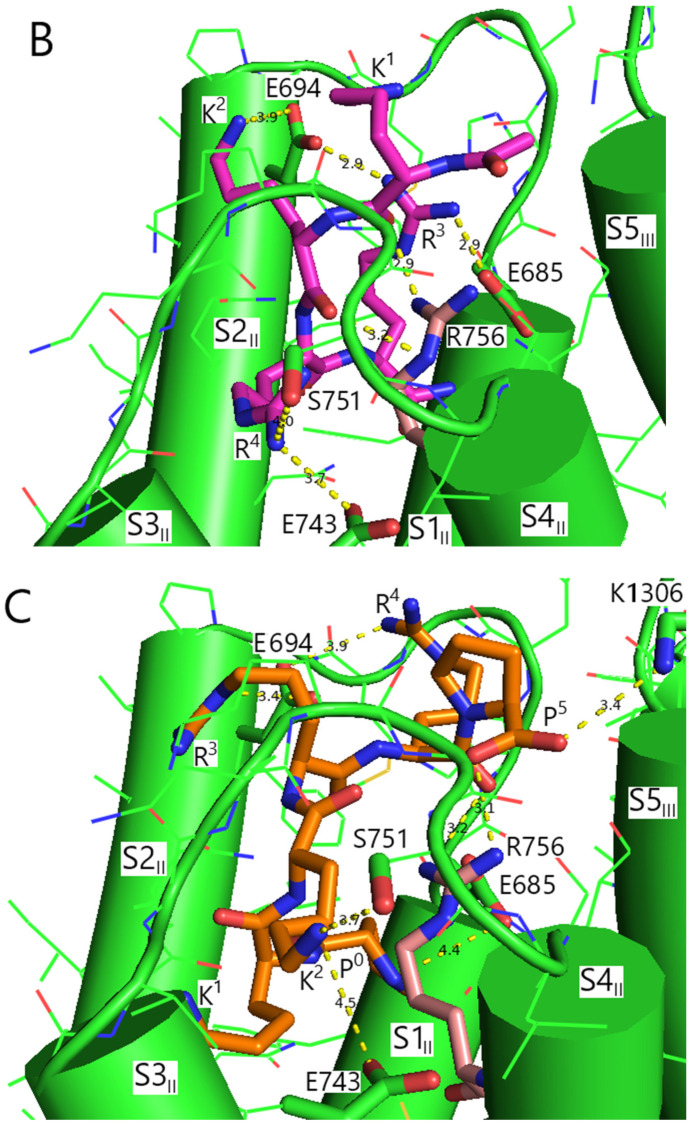
Lateral views of the lowest-energy Ac-KKRR-NH_2_ (**A**,**B**) and H-PKKRRP-OH (**C**) docking poses with site E. The Na_V_1.8 channel is shown as a green cartoon with lines. Cationic residues of the S4_II_ voltage sensor are displayed with pink sticks. Several amino acid residues important for the peptide binding are highlighted with green sticks. Docked peptides are shown in colored sticks. Standard atom coloring is used: oxygen, red; nitrogen, blue. Hydrogen atoms are not presented for clarity. Intermolecular ionic and hydrogen bonds are displayed with yellow dashed lines. Distances between heavy atoms in a bond are given in angstroms.

Figure 13 displays the lowest-energy docking poses of the studied peptides at site E. When the N-end of the Ac-KKRR-NH_2_ molecule is positioned closer to the PD S5_III_ segment (E = −7.1 kcal/mol) (Figure 13A), all positively charged functional groups of the bound peptide form ionic interactions with the VSD_II_ residues: K^1^ and K^2^ interact with E743; R^3^ with E694; and R^4^ with E685. A number of hydrogen bonds are also formed: between the K^2^ and R^3^ backbone carbonyl oxygens and the R756 guanidinium group, and between the K^1^ amino group and the S751 hydroxyl. When the Ac-KKRR-NH_2_ molecule is positioned otherwise (E = −7.0 kcal/mol) (Figure 13B), the K^1^ amino group is not involved in intermolecular interactions. K^2^ forms an ionic bond with E705; R^3^ with E685 and E694; and R^4^ with E743. Hydrogen bonds are formed between the K^1^ and K^2^ backbone carbonyl oxygens and the R756 guanidinium group, and between the R^4^ guanidinium group and the S751 hydroxyl. Again, it is only hydrogen bonds involving the backbone oxygens that directly affect R756.

When binding to site E (E = −8.3 kcal/mol) (Figure 13C), the H-PKKRRP-OH orientation relative to the PD is the opposite to its docking pose detected at site D. The C-end contacts the PD S5_III_ segment. The C-terminal P^5^ carboxylate anion forms ionic bonds with the R756 guanidinium group of the S4_II_ voltage sensor and the K1306 amino group of the PD S5_III_ segment. R^3^ and R^4^ form ionic bonds with E694. The K^2^ side chain amino group is hydrogen bonded to the S751 hydroxyl group, and the K^2^ main chain carbonyl oxygen is hydrogen bonded to the R756 guanidinium group. Also, the formation of two more intermolecular ionic bonds is rather possible: between the P^0^ N-terminal secondary amino group and E685, and between K^2^ and E743.

Site F, located on the extracellular side of the neuron membrane above VSD_III,_ is mainly comprised of the amino acid residues belonging to the PD S5_IV_ and S6_IV_ segments and ECL S1_III_–S2_III_. Only several docked peptide conformations display direct interactions either with the S4_III_ voltage sensor cationic residues (K1247, R1250, R1253, R1256, and R1259) or with anionic E1172 and D1190 that electrostatically stabilize R1250, R1253, and R1256 in the native environment. E1172 forms ionic bonds with R1250 and R1253, while D1190 forms ionic bonds with R1253 and R1256.

**Figure 14:**
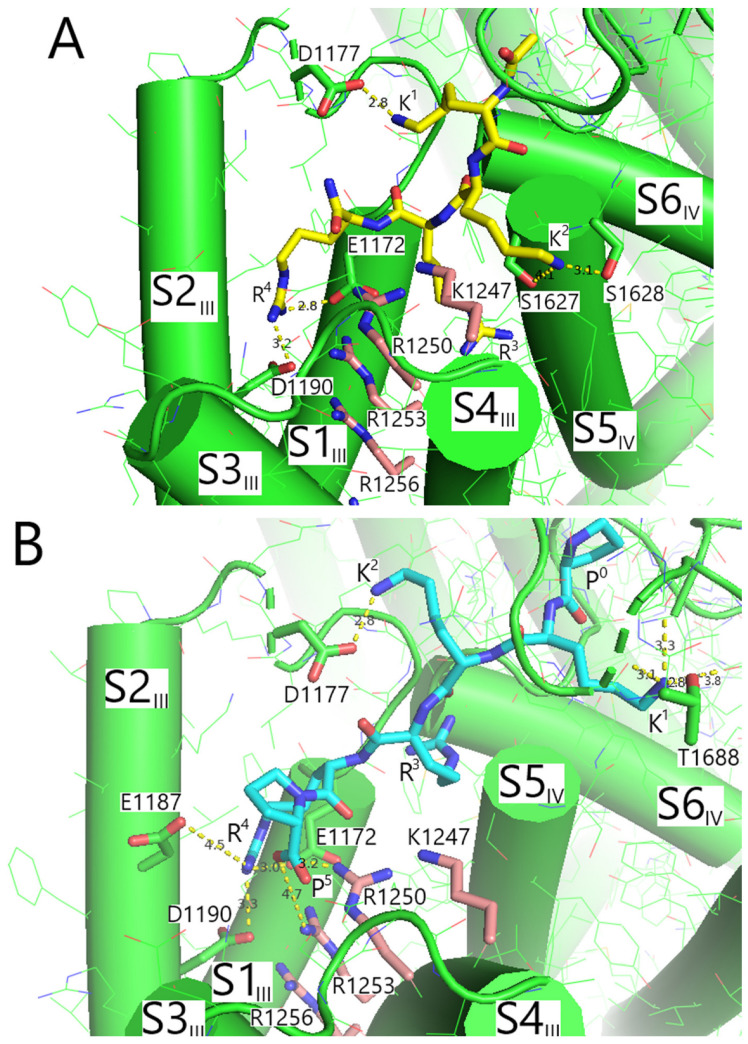

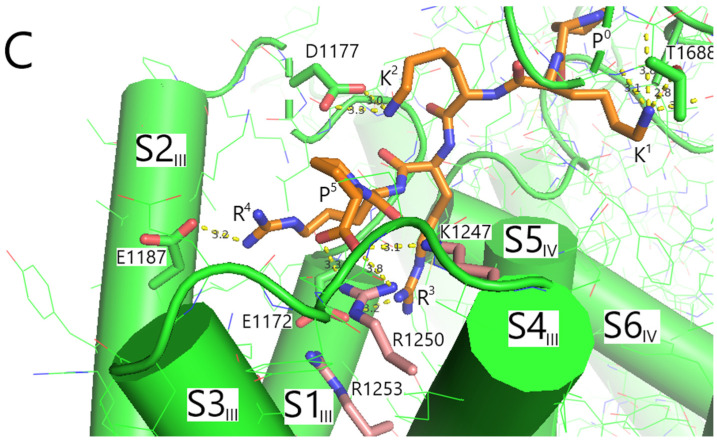
Lateral views of the lowest-energy Ac-KKRR-NH_2_ (**A**) and H-PKKRRP-OH (**B**,**C**) docking poses with site F. The Na_V_1.8 channel is shown as a green cartoon with lines. Cationic residues of the S4_III_ voltage sensor are displayed with pink sticks. Several amino acid residues important for the peptide binding are highlighted with green sticks. Docked peptides are shown in colored sticks. Standard atom coloring is used: oxygen, red; nitrogen, blue. Hydrogen atoms are not presented for clarity. Intermolecular ionic and hydrogen bonds are displayed with yellow dashed lines. Distances between heavy atoms in a bond are given in angstroms.

The 4 lowest-energy Ac-KKRR-NH_2_ ligand–receptor complexes out of 32 obtained are characterized by the deeper protrusion of the R^4^ guanidinium group into the cleft between the S1_III_/S2_III_ and S3_III_/S4_III_ segments. This allows the indicated group to form ionic bonds with both E1172 and D1190, the residues that compensate for the S4_III_ voltage sensor charge (E = −7.6 kcal/mol) (Figure 14A). At the same time, another ionic bond is formed between K^1^ and D1177, while K^2^ is hydrogen bonded to S1627 and S1628 that belong to the PD S5_IV_ segment. The S4_III_ voltage sensor residues are not affected at all, even with hydrogen bonds.

On the contrary, only 8 out of 23 H-PKKRRP-OH conformations do not display any intermolecular ionic bonds with the S4_III_ residues, while the other 15 ligand–receptor complexes contain at least one such bond, between the P^5^ C-terminal carboxylate anion and R1250. In 10 ligand–receptor complexes out of 15, the C-terminal P^5^ carboxylate anion simultaneously interacts with two cationic S4_III_ residues, either with R1250 and R1253 (5) or with K1247 and R1250 (5). The corresponding lowest-energy H-PKKRRP-OH docking poses (E = −8.2 and −8.1 kcal/mol) are presented in Figure 14B,C. In both cases, K^1^ forms a cluster of hydrogen bonds with the residues belonging to the ECL that precedes the S6_IV_ segment, the T1688 side chain hydroxyl group, and the glycine G1687, G1690, and cysteine C1692 backbone carbonyl oxygens, while K^2^ electrostatically interacts with D1177. When the P^5^ carboxylate anion forms ionic bonds with R1250 and R1253, R^4^ is bound to E1172, E1187, and D1190 (Figure 14B). When the P^5^ carboxylate anion forms ionic bonds with K1247 and R1250, R^3^ is bound to E1172, and R^4^ is bound to E1187 (Figure 14C).

### 2.5. The Formalin Test

**Figure 15:**
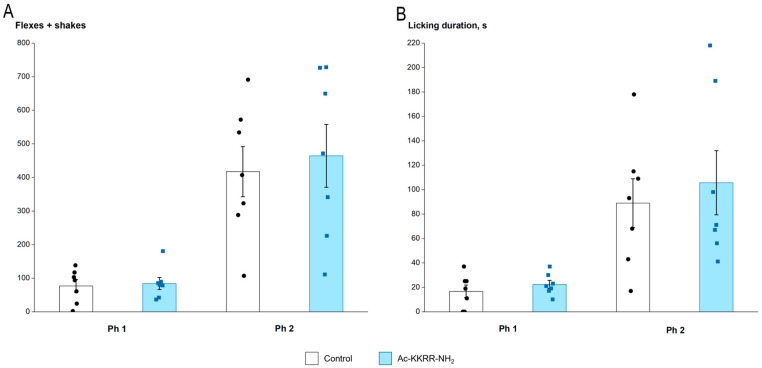
Effect of Ac-KKRR-NH_2_ on the number of flexes + shakes and licking duration in the formalin test. (**A**) The number of flexes + shakes in the acute (Ph1) and tonic (Ph2) phases; (**B**) licking duration in the acute (Ph1) and tonic (Ph2) phases. Data are presented as mean ± SEM.

In the first set of experiments (Figure 15), the Ac-KKRR-NH_2_ administration (10.0 mg/kg) did not result in significant differences in the number of flexes and shakes compared with control in either the acute (84.3 ± 17.8 vs. 77.0 ± 18.9, *p* = 0.783, *t* = −0.280) or tonic phases (464.5 ± 74.7 vs. 417.4 ± 74.7, *p* = 0.701, *t* = −0.393). Similarly, no significant differences in licking duration were observed between experimental and control animals in the acute (22.4 ± 3.3 vs. 16.7 ± 5.2 s, *p* = 0.374, *t* = −0.922) or tonic phase (105.7 ± 26.3 vs. 89.0 ± 19.9 s, *p* = 0.621, *t* = −0.506).

**Figure 16:**
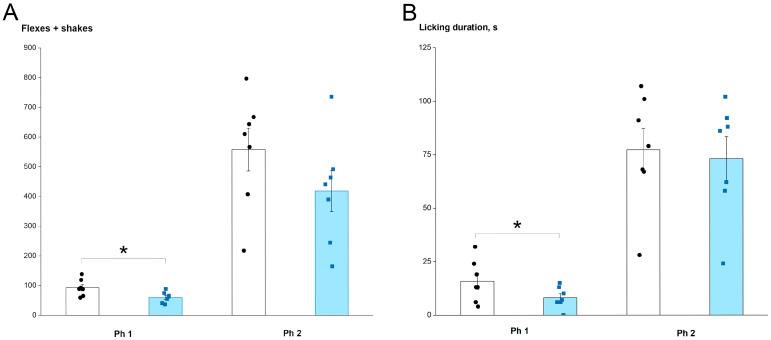
Effect of H-PKKRRP-OH on the number of flexes + shakes and licking duration in the formalin test. (**A**) The number of flexes + shakes in the acute (Ph1) and tonic (Ph2) phases; (**B**) licking duration in the acute (Ph1) and tonic (Ph2) phases. Data are presented as mean ± SEM. Statistically significant difference between the control and experimental values is designated with the asterisk (* *p* < 0.05).

In the second set of experiments (Figure 16), the H-PKKRRP-OH administration (10.0 mg/kg) significantly reduced the number of flexes and shakes in the acute phase compared with control (60.0 ± 6.9 vs. 93.0 ± 10.6, *p* = 0.023, *t* = −2.614). In the tonic phase, a decrease that did not reach statistical significance was observed (418.0 ± 69.7 vs. 556.5 ± 52.4, *p* = 0.077, *t* = 1.932). At the supraspinal level, licking duration was significantly reduced in the acute phase (8.1 ± 1.9 vs. 16.7 ± 3.4 s, *p* = 0.045, *t* = 2.226), whereas no significant differences were observed in the tonic phase (73.1 ± 10.2 vs. 77.3 ± 10.1 s, *p* = 0.771, *t* = 0.289).

Thus, Ac-KKRR-NH_2_ at 10.0 mg/kg did not significantly alter nociceptive behavior at both the spinal and supraspinal levels. In contrast, the same dose of H-PKKRRP-OH reduced nociceptive responses in the acute phase at both levels, with no significant effect in the tonic phase.

**Figure 17:**
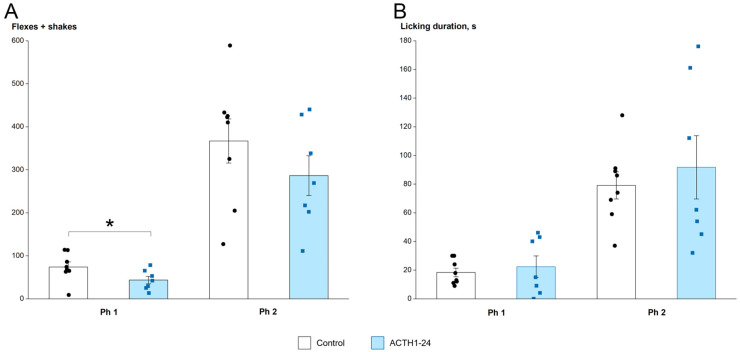
Effect of ACTH(1–24) on the number of flexes + shakes and licking duration in the formalin test. (**A**) The number of flexes + shakes in the acute (Ph1) and tonic (Ph2) phases; (**B**) licking duration in the acute (Ph1) and tonic (Ph2) phases. Data are presented as mean ± SEM. Statistically significant difference between the control and experimental values is designated with the asterisk (* *p* < 0.05).

Finally, the ACTH(1–24) administration at 2.5 mg/kg resulted in a decrease in the number of flexes and shakes in the acute phase (Figure 17) compared with control (43.6 ± 8.8 vs. 77.4 ± 11.8, *p* = 0.043, *t* = −2.234). No significant differences were observed in the tonic phase (286.4 ± 46.1 vs. 367.0 ± 51.3, *p* = 0.269, *t* = 1.153). At the supraspinal level, licking duration did not differ significantly between the groups in either the acute (22.4 ± 7.5 vs. 18.4 ± 3.0 s, *p* = 0.610, *t* = −0.526) or tonic phase (91.7 ± 22.0 vs. 79.1 ± 9.4 s, *p* = 0.590, *t* = −0.550).

## 3. Discussion

The experimental part of our work yielded unique data on the behavior of a special substructure of the living molecule, the Na_V_1.8 channel activation gating system, under intact conditions. The key parameter obtained is Z_eff_, the effective charge transferred by the Na_V_1.8 channel activation gating system upon the channel opening, which determines the nociceptive neuron excitability in vivo and primarily controls the peripheral nerve impulse coding that is perceived as a pain sensation at the organismal level [32].

It has been demonstrated in the current study that a comprehensive methodological approach, from the atomic and molecular levels of consideration to behavioral experiments, can enable the creation of new effective analgesics. This approach could be implemented due to the specific Na_V_1.8 channel properties. First, these channels are markers of nociceptive neurons [23], thus confirming that our experiments have been conducted on an identified sensory neuron. Second, the Na_V_1.8 channel steady-state activation and inactivation characteristics are separated along the voltage (E) axis in such a way that the amplitude values of the sodium current at a fixed E (close to its threshold values) are achieved when the activation gating system is in the open state, while the inactivation process has not yet started. Therefore, when recording the Na_V_1.8 channel chord conductance function G_Na_(E)^norm^ (Figure 4), we register the response of the Na_V_1.8 activation gating system only, unaffected by the inactivation process. In the classical Hodgkin–Huxley Nav1.1 channel, the activation process interferes with the inactivation process, making it impossible to correctly estimate the Z_eff_ value using the Almers method [32].

To accurately evaluate Z_eff_, a strict control of the series resistance Rs is required. According to the findings in the field of voltage-clamp theory, which is currently receiving little attention, Rs is the key parameter that determines not only the steady-state error of the method but also the kinetic characteristics of the gating systems [33]. In our case, if Rs exceeds 3 MΩ (this characteristic directly depends on the resistance of the patch electrode), then a correct construction of the logarithmic sensitivity function L(E) and, of course, Z_eff_ evaluation becomes impossible. Thus, the Almers–Timin method implemented herein has provided a unique opportunity to obtain the Z_eff_ values and quantitatively describe the mechanisms of ligand–receptor binding, where intermolecular ionic interactions play a critical role.

Our patch-clamp results indicate that ACTH and H-PKKRRP-OH at 100 nM function as Na_V_1.8 gating modulators rather than channel blockers. Hence, the observed effects are likely mediated by peptide binding to VSDs from the extracellular side, which should obstruct the intramembrane movement of the S4 voltage sensors or even trap these segments in a specific conformation. However, Ac-KKRR-NH_2_ at the same concentration does not modulate the Na_V_1.8 channel functional activity, despite the fact that both studied short peptides share the same cationic KKRR motif and dock with VSD_I_–VSD_III_ (sites D–F). Docking helps reveal the differences between the Ac-KKRR-NH_2_ and H-PKKRRP-OH binding mechanisms that account for the absence of the effect of Ac-KKRR-NH_2_.

It is also of importance to compare the results obtained herein with the earlier studied mechanism of the cationic tripeptide Ac-KKK-NH_2_ binding to the Na_V_1.8 channel molecule [28]. This tripeptide decreases the Z_eff_ value in the patch-clamp experiments at 100 nM and demonstrates a strong analgesic effect in the formalin test at 1.0 mg/kg. The Ac-KKK-NH_2_ molecule binds exclusively to VSD_I_ (site D), displaying no effective docking with VSD_II_ and VSD_III_ (sites E and F). The D151, E157, T161, R218, and R221 amino acid residues of VSD_I_ have been identified as the putative contact residues upon the Ac-KKK-NH_2_ binding. Two lysine side chain amino groups form intermolecular ionic bonds with D151 and E157. These anionic residues compensate for the positive charge of R215 that belongs to the S4_I_ voltage sensor. The third Ac-KKK-NH_2_ amino group is involved in a hydrogen bond with T161 and cation-π interactions with two S4_I_ voltage sensor residues, R218 and R221. Thus, the ligand–receptor interactions resulting from the Ac-KKK-NH_2_ binding have been demonstrated to be able to directly affect the S4_I_ conformational freedom.

As opposed to both Ac-KKK-NH_2_ and H-PKKRRP-OH, the Ac-KKRR-NH_2_ molecule is not shown to form any ionic interactions with the VSD_I_ residues, including the anionic D151 and E157, and its docking pose at site D is mainly stabilized with intermolecular hydrogen bonds (Figure 12). The H-PKKRRP-OH hexapeptide forms two direct ionic bonds with R218 and R221 belonging to the S4_I_ voltage sensor, as well as another three with the S2_I_ D151 and E157 residues. In addition to the VSD_I_ residues, the H-PKKRRP-OH molecule also interacts with Q807 of the PD S5_II_ segment. Thus, the H-PKKRRP-OH binding at site D might restrict the movement of the entire VSD_I_ relative to the PD. It is also likely that due to direct H-PKKRRP-OH interaction with R218 and R221, the S4_I_ segment can be trapped in the observed conformation, which should obstruct the movement of the S4_I_ voltage sensor up and down within the neuron membrane as VSD_I_ responds to changes in membrane potential during the Na_V_1.8 channel working cycle.

When docked at site E (Figure 13), the Ac-KKRR-NH_2_ molecule does not directly interact with the PD residues. All positively charged Ac-KKRR-NH_2_ functional groups are involved in ionic bonds with the S1_II_–S3_II_ residues of VSD_II_ (E685, E694, and E743), but this should barely affect the conformational freedom of the S4_II_ voltage sensor cationic residues. The H-PKKRRP-OH molecule interacts with E685, E694, and E743 as well, but it also forms ionic bonds with both the S4_II_ voltage sensor and the PD S5_III_ residues, which indicates that H-PKKRRP-OH might trap the S4_II_ voltage sensor in the ligand–receptor complex. It should be noted, though, that the H-PKKRRP-OH binding to site E directly involves only one of the five S4_II_ cationic residues, the most sterically accessible residue R746, whereas its binding to site D can directly involve three of the four S4_I_ voltage sensor residues: R215, R218, and R221.

Peptide docking at site F (Figure 14) is characterized by the absence of intermolecular hydrogen bonds with the participation of backbone carbonyl oxygens of the studied molecules. One of the Ac-KKRR-NH_2_ lysine amino groups (K^2^) is hydrogen bonded to the S1627 and S1628 hydroxyls (PD S5_IV_ segment). Other Ac-KKRR-NH_2_ cationic moieties (K^1^ and R^4^) form ionic bonds with E1172, D1177 (ECL S1_III_–S2_III_), and D1190 (S2_III_). The latter two anionic residues electrostatically interact with R1250, R1253, and R1256 of the S4_III_ voltage sensor. Similar to Ac-KKRR-NH_2_, the H-PKKRRP-OH molecule forms ionic bonds with E1172 (R^4^), D1177 (K^2^), and E1187 on the S2_III_ segment (R^3^ or R^4^, depending on the docking pose) and sometimes D1190 (R^4^, also depending on the docking pose). One of its lysine amino groups (K^1^) is also hydrogen bonded to the PD, but to the G1687, T1688, G1690, and C1692 residues that belong to the ECL preceding the S6_IV_ segment. In addition, the H-PKKRRP-OH P^5^ C-terminal carboxylate anion forms two direct ionic bonds with the S4_III_ cationic residues: R1250 and either K1247 or R1253. Therefore, the S4_III_ voltage sensor of VSD_III_ can be trapped as a result of the H-PKKRRP-OH binding.

The docking results indicate that the H-PKKRRP-OH ability to form direct ionic bonds with the cationic residues of the voltage sensors (S4) upon binding to the Na_V_1.8 channel might be crucial for its analgesic effect. This becomes possible because the H-PKKRRP-OH molecule contains the anionic C-terminal carboxylate moiety, as opposed to Ac-KKRR-NH_2_. In addition, an extensive network of ionic and hydrogen bonds between the H-PKKRRP-OH cationic functional groups and the amino acid residues of other VSD (S1–S3) and PD (S5–S6) segments provides the complementarity of the H-PKKRRP-OH molecule to its predicted Na_V_1.8 channel binding sites. The following amino acids are identified by docking to be the putative contact residues upon the H-PKKRRP-OH binding, as they are involved in direct intermolecular ionic interactions with the peptide: VSD_I_ (site D), D151, E157, R218, and R221; VSD_II_ (site E), E685, E694, E743, and R746; VSD_III_ (site F), E1172, D1177, E1187, D1190, K1247, R1250, and R1253. Importantly, the same amino acid residues have been shown to participate in the Ac-KKK-NH_2_ binding with VSD_I_ [28].

Thus, the H-PKKRRP-OH molecule in the ligand–receptor complex with the Na_V_1.8 channel might serve as a linker that strengthens the interaction of the S4 voltage sensors with the S1–S3 segments within VSD_I_-VSD_III_, also possibly attaching the VSDs to the PD. Our calculations demonstrate a weaker H-PKKRRP-OH binding to VSD_II_, where the attacking peptide forms just one direct ionic bond with the most sterically accessible cationic residue of the S4_II_ voltage sensor, as opposed to the H-PKKRRP-OH binding to VSD_I_ and VSD_III_.

It is tempting to suggest that an analgesic short cationic peptide should contain a negatively charged functional group, which would provide direct ionic interactions with the S4 voltage sensor residues. However, the earlier discovered analgesic effect of the Ac-KKK-NH_2_ tripeptide [28] containing no anionic moieties indicates that the intermolecular interactions, which result in a decrease in the Na_V_1.8 channel voltage sensitivity, can be of another origin than ionic bonds. In the case of Ac-KKK-NH_2_, it is likely the cation-π interactions of an Ac-KKK-NH_2_ lysine amino group and two arginine guanidinium groups of the S4_I_ voltage sensor. Despite the apparent similarity in the amino acid sequences of Ac-KKK-NH_2_ and Ac-KKRR-NH_2_, according to our results, the cationic functional groups of the latter peptide do not interact directly with the S4 residues, which would account for the absence of the Ac-KKRR-NH_2_ effect in the patch-clamp experiments and in the formalin test. Seemingly, it is electrostatically complicated to dock four positively charged moieties of the attacking peptide in a pose that favors any direct ligand–receptor interactions with the cationic S4 voltage sensor residues. Indeed, Ac-KKRR-NH_2_ has not been shown to form intermolecular ionic bonds and cation-π interactions with the S4_I_ residues upon binding to VSD_I_, where specifically docks the Ac-KKK-NH_2_ tripeptide. The H-PKKRRP-OH C-terminal anionic group partially facilitates the electrostatic clash, providing an opportunity to form direct salt bridges with the positively charged voltage sensor residues.

It should be noted that all average values of intramolecular distances between the cationic functional groups in both non-analgesic Ac-KKRR-NH_2_ and analgesic H-PKKRRP-OH molecules obtained by conformational analysis fall in the range of 9–12 Å, earlier suggested to be characteristic for the short cationic analgesic peptides [25,26,27,28], except for the K^1^–R^4^ distance in the Ac-KKRR-NH_2_ molecule. Hence, direct blind docking of the attacking peptides with the Na_V_1.8 channel molecule is required to predict their ability to modulate the Na_V_1.8 channel functioning. However, conformational analysis remains necessary to provide the steric and energetical criteria for the selection of peptide conformations for further docking.

It should also be noted that the mechanistic conclusions presented above cannot be demonstrated by docking alone. Docking can support a structural hypothesis, but establishing residue essentiality or a causal gating mechanism would require additional validation, such as mutagenesis, electrophysiology on mutant channels, molecular dynamics simulations, rescoring, or free-energy calculations. We have to make it clear that a single cryo-EM-derived Na_V_1.8 structure as a static receptor model was used herein. Since the proposed mechanism depends on specific residue-level contacts with VSDs, it should be explicitly acknowledged that docking on cryo-EM ion channel structures can be affected by local structural uncertainty [34,35].

Another issue to be addressed is the species consistency. The electrophysiological experiments were performed in rat DRG neurons, whereas docking was carried out using the human Na_V_1.8 structure. The amino acid sequences of the entire structural segments of human and rat Na_V_1.8 channels (UniProt IDs Q9Y5Y9 and Q62968, respectively) that constitute the predicted binding sites D-F of the studied short ACTH-related cationic peptides are aligned in Table 3. The data presented demonstrate a great extent of homology, especially within VSD_I_, which suggests that the patch-clamp data obtained in rats could be translated into humans. The residues involved in intermolecular ionic and hydrogen bonds between the docked peptides and the human Na_V_1.8 structure are mostly conserved between the species, with a few minor exceptions (T204/A204, E694/D693, D1177/E1178, S1627/A1628, and T1688/S1689). Only two of these sequence differences might affect the intermolecular bonds formed between the peptide molecules and the Na_V_1.8 channel: the ligand–receptor hydrogen bonds with the participation of the T204 and S1628 residues, which are not considered critical for effective peptide binding. All intermolecular ionic bonds should be retained irrespective of the species, which indicates that the docking results obtained in the human Na_V_1.8 model are consistent with the experimental results obtained in rats.

The analgesic effect of H-PKKRRP-OH predicted by the results of our patch-clamp experiments and theoretical blind docking is confirmed in vivo in the formalin test. The organotypic tissue culture method demonstrates the H-PKKRRP-OH neurite-inhibiting effect at 10 μM in the DRG explants, as opposed to both ACTH and Ac-KKRR-NH_2_ at the same concentration, which suggests that H-PKKRRP-OH might control neurite growth by a receptor-mediated mechanism. This suggestion needs further verification, as the current data do not distinguish between a specific receptor-mediated effect, nonspecific growth inhibition, or toxicity. The H-PKKRRP-OH molecule has been designed by us to contain the proline residues at both ends, thus protecting the peptide from proteolytic decomposition during delivery to its molecular target, the Na_V_1.8 channel in the nociceptive neuron membrane. Taken together, these facts indicate that H-PKKRRP-OH might be a promising candidate for a role as an analgesic medicinal substance, effective for the correction of pathological states accompanied by uncontrolled nerve tissue growth.

It should be noted that full-length ACTH was used in patch-clamp experiments, whereas the in vivo formalin test was performed with ACTH(1–24). This choice was based on structural and functional considerations underlying our working hypothesis. According to our model, the interaction of ACTH with the Na_V_1.8 channel is primarily mediated by its cationic region containing the KKRR motif. Notably, this sequence is fully preserved within the ACTH(1–24) fragment. Also, ACTH(1–24) is known to retain the principal biological activity of the full-length hormone and is widely used as a functionally relevant ACTH mimetic in experimental and clinical settings [36,37]. Importantly, truncation of the C-terminal region by 15 amino acid residues reduces conformational flexibility of the molecule, which may facilitate more specific interactions with membrane targets such as ion channels. Therefore, ACTH(1–24) can be regarded as a structurally and functionally relevant ACTH-based peptide that retains the putative Na_V_1.8-interacting region while substantially reducing the conformational complexity associated with the full-length molecule. The use of this fragment in the formalin test allowed us to specifically assess whether the N-terminal cationic region of ACTH is sufficient to produce an antinociceptive effect at the organismal level. The observed reduction in nociceptive responses following ACTH(1–24) administration supports this assumption.

The direct modulation of the Na_V_1.8 channel voltage sensitivity detected in patch-clamp experiments indicates the third ACTH antinociceptive mechanism, in addition to the earlier studied ACTH-induced analgesic effects associated with activation of opioid receptors and MC2R receptor-mediated production of glucocorticoids [22]. The cited results were obtained in anesthetized rats, which means that the Na_V_1.8 channels had been blocked. Very likely, the ACTH(4–7) MEHF motif is involved in activation of opioid receptors because the commercially available ACTH-related Semax peptide (MEHFPGP) has been recently demonstrated to function as a μ-opioid receptor agonist [38]. To activate the MC2R receptor controlling glucocorticoid biosynthesis in the adrenal gland, both ACTH(6–9) HFRW and ACTH(15–19) KKRRP fragments are required [39,40]. As the H-PKKRRP molecule contains neither the MEHF nor the HFRW motifs, the above literature data support the idea that the analgesic properties of ACTH- and ACTH-related peptides discussed herein may be at least partially attributed to their direct interaction with the Na_V_1.8 channel via the cationic KKRR motif.

Despite full-length ACTH being too long and flexible for informative docking, the data presented herein make it possible to speculate that ACTH, ACTH(1–24), and H-PKKRRP-OH ligand–receptor binding is targeted at the VSD_I_-VSD_III_ extracellular sides, thus shielding the charges transported by the voltage sensors within the nociceptive neuron membrane during the Na_V_1.8 channel working cycle. Direct ligand–receptor interactions that involve the cationic residues of a voltage sensor seem to be crucial to effectively modulate its functioning. In the patch-clamp experiments, this effect is manifested in a decrease in the effective charge carried by the Na_V_1.8 channel activation gating system upon the channel opening, Z_eff_. Considering the nanomolar range of concentrations, it does not seem probable that more than one attacking peptide molecule actually binds to a Na_V_1.8 channel at the same time. It cannot be unambiguously indicated which particular VSD is modulated due to the ligand binding, as all three VSD_I_-VSD_III_ domains belong to the activation gating system. However, the docking results obtained herein and earlier [28] allow us to propose the following targeting order: VSD_I_ > VSD_III_ > VSD_II_. Suggestively, the VSD modulation by the attacking peptides decreases the lifetime of a single Na_V_1.8 channel in the open state, which is manifested in an analgesic effect at the organismal level. This mechanism is fundamentally different from the effect of Na_V_1.8 channel blockers and well deserves further investigation.

The Na_V_1.8 channels can be regarded as promising targets for the development of novel analgesics. They play a key role in the generation of action potentials in primary sensory neurons, determining the subtle pattern of nociceptor impulse firing [41,42]. Modulating (rather than blocking) the Na_V_1.8 channel functional activity, it becomes possible to specifically switch off only the pronociceptive, higher-frequency component of the impulse firing that informs the central nervous system about the damaging stimulus. At the same time, the lower-frequency component of the impulse firing of polymodal nociceptors, signaling about an adequate (e.g., tactile or thermal) stimulus, remains unaffected. Therefore, a specific modulation of the Na_V_1.8 channel voltage sensitivity, which reduces the nociceptive neuron excitability, allows for the transmission of other vitally important information to the central nervous system. As a result, the analgesics we are developing may be particularly in demand for the relief of chronic pain when long-term use of analgesic drugs is required. The effect of local anesthetics that completely block sodium channels also totally interrupts the communication between polymodal nociceptors and the central nervous system, which, with prolonged use of these drugs, dramatically reduces the quality of life of patients suffering from chronic pain. The Na_V_1.8 channels are known to play an important role in hyperexcitability and pain caused by damage to somatic structures [43,44,45]. The amplitude of the Na_V_1.8 currents in sensory neurons is increased by a number of mediators, such as adenosine [46], nerve growth factor [47], etc. It has been found that the density of Nav1.8 channels in nociceptors increases with severe toothache [48]. It is also known that Na_V_1.8 channels are responsible for the manifestations of neuropathic pain, for example, in diabetic neuropathy [49]. An increase in the Na_V_1.8 channel functional activity is noted in cases of mechanical damage, inflammatory processes, and the action of agents that cause hyperalgesia. A decrease in this activity, leading to an antinociceptive effect, can be carried out in two ways: by reducing the Na_V_1.8 channel density in the nociceptive neuron membrane or by reducing the voltage sensitivity of the Na_V_1.8 channel activation gating system [46,50,51,52]. Apparently, the agents capable of reducing the excitability of nociceptors by reducing the Na_V_1.8 channel voltage sensitivity can claim the role of analgesic medicinal substances, the effect of which should be rather attenuated upon their long-term use.

Stress triggers neurohumoral processes in the human brain, which involve the nociceptive system functioning. One such mechanism has been described in the present work at the molecular level of consideration. It has to be noted, though, that statistically significant analgesic effects of H-PKKRRP-OH (10.0 mg/kg) and ACTH(1–24) (2.5 mg/kg) were detected only in the first, acute phase of the formalin test (Figure 16 and Figure 17). This raises questions regarding the duration of target coverage and whether sufficient concentrations have been maintained throughout the experiment. In the absence of pharmacokinetic and human Na_V_1.8 electrophysiological data, it remains difficult to determine whether the limited efficacy observed in the current study reflects insufficient exposure, rapid peptide degradation, or suboptimal target engagement. Given that another two previously investigated short cationic peptides protected against proteolysis, Ac-KEKK-NH_2_ (3.0 mg/kg) and Ac-KKK-NH_2_ (1.0 mg/kg), have demonstrated significant analgesic effects in both acute and tonic phases of the formalin test [27,28] in the similar range of concentrations, suboptimal target engagement appears to be the most feasible explanation for the lack of the H-PKKRRP-OH and ACTH(1–24) analgesic effects in the tonic phase.

The second, tonic phase of the formalin test is determined by the inflammatory agents released in response to formalin, i.e., prostaglandins, bradykinin, histamine, and serotonin, which cause sensitization of primary and spinal sensory neurons and the inflammatory process. The central sensitization includes immune cells, central microglia, and sex hormones. Currently, physiology pays special attention to the immune system, in particular, microglia. Microglial cells surround each neuron in the brain and ensure its normal functioning for signal transmission. Pain activates immune microglia, which secrete inflammatory mediators, cytokines that affect the synaptic plasticity of brain neurons. Thus, the nerve cell conducts the signal, and the microglia ensures its functioning. It is possible that under our conditions, both H-PKKRRP-OH and ACTH(1–24) do not sufficiently reduce proinflammatory cytokines to display a significant protective antinociceptive effect during the tonic phase, though a decrease in the number of flexes and shakes (Figure 16 and Figure 17) suggests a possible analgesic effect at the spinal level in the tonic phase of the formalin test.

It should be emphasized that the main result of the present study is the discovery of a novel physiological mechanism linking activation of the HPA axis with the nociceptive system of the brain at the molecular level. Future experiments will make it possible to investigate the suggested mechanism in more detail, which should result in the creation of new safe analgesic medicinal substances of peptide nature.

## 4. Materials and Methods

### 4.1. Chemicals and Reagents

Except for the short peptides, all chemicals used in the experiments were from Sigma-Aldrich (St. Louis, MA, USA). The short peptides Ac-KKRR-NH_2_ and H-PKKRRP-OH were synthesized in the Verta Research and Production Company (St. Petersburg, Russia) by the method of classic peptide synthesis using reagents from Sigma-Aldrich and Iris Biotech GmbH (Marktredwitz, Germany). Newly synthesized short peptides were characterized with high-performance liquid chromatography (purity ≥ 95%) and mass spectrometry.

### 4.2. Dissociated Sensory Neuron Culture

Dissociated sensory neurons were isolated from dorsal root ganglia (DRG) located in the L5–S1 region of Wistar rats (P3–P5) using a short-term culture protocol. Neurons were obtained from 15 independent culture preparations; the total number of animals used was 15. After isolation, the ganglia were placed in Hanks’ solution and subjected to enzymatic treatment at 37 °C for 5–8 min, depending on the age of the animals. The enzymatic treatment solution (pH = 7.4) contained Hanks’ solution (1 mL), Eagle’s medium (1 mL), collagenase type 1A (2 mg/mL), pronase E (1 mg/mL), and Na-HEPES (1 mM). After the treatment, the ganglia were centrifuged for 1 min at 900 rpm, and the supernatant solution was removed. The ganglia were then resuspended in Eagle’s medium with the addition of fetal bovine serum (10%), glucose (0.6%), gentamicin (40 U/mL), and L-glutamine (2 mM). Mechanical dissociation of sensory neurons was carried out by pipetting, and the cell suspension was further diluted with culture medium until the required density of neurons was obtained. Visible fragments of connective tissue were manually removed using a micropipette. Isolated sensory neurons were cultured in 40 mm collagen-coated Petri dishes at 37 °C and 5% CO_2_ in a CO_2_ incubator (Sanyo Co., Ltd., Fujioka, Japan) for 1–2 h and further used to record the Na_V_1.8 sodium currents.

### 4.3. Experimental Solutions

In the patch-clamp experiments, the experimental bath with the dissociated nociceptive neurons was filled with the extracellular solution, while the patch pipettes were filled with the intracellular solution. The extracellular solution (pH = 7.4) contained choline chloride (70 mM), NaCl (65 mM), HEPES-Na (10 mM), CaCl_2_ (2 mM), MgCl_2_ (2 mM), and tetrodotoxin (100 nM). The intracellular solution (pH = 7.2) contained CsF (100 mM), CsCl (40 mM), NaCl (10 mM), HEPES-Na (10 mM), and MgCl_2_ (2 mM). The pH values were adjusted with NaOH and HCl solutions. The chosen experimental solutions made it possible to selectively record the Na_V_1.8 sodium currents. In the absence of potassium ions, all potassium currents were excluded. Calcium currents were suppressed by fluoride ions. Tetrodotoxin was introduced into the extracellular solution to block tetrodotoxin-sensitive sodium channels. When the responses of the slower tetrodotoxin-resistant Na_V_1.9 channels were visually identified in the course of an experiment based on their characteristic kinetics, recordings were terminated.

### 4.4. Registration of Na_V_1.8 Currents

The patch-clamp method was applied in the whole-cell configuration using a hardware-software complex that included an L/M-EPC 7 amplifier (HEKA Elektronik, Reutlingen, Germany), analog-to-digital and digital-to-analog converters, and a personal computer with custom-developed software to automate the experiments.

Microelectrodes with 1–2 MΩ resistance were made of borosilicate glass using a P-97 micropipette puller (Sutter Instrument, Novato, CA, USA) [53]. Using a micromanipulator, a pipette filled with intracellular solution was brought to the neuron surface to establish a gigaseal contact with the membrane. To implement the “whole-cell” configuration, the membrane section under the pipette was ruptured by applying negative pressure. After the gigaseal formation, the neuron was lifted above the bottom of the experimental chamber using a micromanipulator to minimize noise and ensure free flow of extracellular solution. Recordings of sodium currents began a few minutes after the whole-cell access had been established, allowing for the intracellular dialysis. The series resistance value (R_S_), determining both the dynamic and steady-state errors of the patch-clamp method [33], was constantly monitored during an experiment. The recordings were continued for at least 15 min to allow equilibration of peptide–channel interactions and terminated if R_S_ exceeded 3 MΩ. The maximum duration of the experiment was 60 min.

When running the experiment, a sequence of 50 ms voltage steps from −60 to +50 mV was applied to the neuron membrane in 5 mV increments. The amplitude values of the sodium current were used to construct the current–voltage function I_ampl_(E) and the voltage dependence of the chord conductance G_Na_(E). To determine the effective charge of the Na_V_1.8 channel activation gating system (Z_eff_, in elementary charge units), the modified Almers method was implemented [41].

To assess the effects of ACTH, H-PKKRRP-OH, and Ac-KKRR-NH_2_ on the Na_V_1.8 channel functional activity, the studied substances were applied to the experimental bath at 100 nM. Each peptide was tested on 20 cells. For every cell, control recordings were first obtained under baseline conditions, after which the corresponding peptide was applied, and its effects were assessed.

### 4.5. The Modified Almers Method

Unlike fast Na_V_1.1 channels, the Na_V_1.8 channels are characterized by a rightward shift of the inactivation curve along the voltage axis. Hence, the amplitudes of the Na_V_1.8 currents are primarily determined by the activation gating system, while the inactivation variable is close to unity. For Na_V_1.8 channels, it is possible to apply the modified Almers method, which makes it possible to obtain reliable Z_eff_ values using the amplitude values of the currents [32].

The ratio of the number of open (N_O_) to the number of closed (N_C_) Na_V_1.8 channels was calculated as follows:

$$\frac{N_{O}}{N_{C}}=\frac{G_{Na}(E)}{G_{Na}^{max}-G_{Na}(E)},$$

where G_Na_(E) is the voltage dependence of the chord conductance, and $G_{Na}^{max}$ is the maximum value of the G_Na_(E) function. The G_Na_(E) function was constructed from the experimental patch-clamp data:

$$G_{Na}\left(E\right)=\frac{I_{ampl}(E)}{E-E_{Na}},$$

where I_ampl_(E) is the amplitude value of the Na_V_1.8 current, and E_Na_ is the reversal potential of the Na_V_1.8 current.

G_Na_(E) is a monotonic function approaching its maximum value $G_{Na}^{max}$ at positive E. In this case, it was in agreement with the Almers theory:

$$\underset{E\to -\infty }{\lim}(\frac{N_{O}}{N_{C}})=\underset{E\to -\infty }{\lim}\left(\frac{G_{Na}\left(E\right)}{G_{Na}^{max}-G_{Na}\left(E\right)}\right)\underset{E\to -\infty }{\to }const\cdot \exp\left(\frac{Z_{eff}\cdot e_{0}\cdot E}{k\cdot T}\right),$$

where k is the Boltzmann constant, T is the absolute temperature, const is a constant, and e_0_ is the electron charge.

When the membrane potential tends to approach negative infinity (E → −∞), Z_eff_ can be evaluated from the slope of the asymptote passing through the first points determined by negative E values. At these potentials, the limit of the function under study tends to one-exponential dependence described by the Boltzmann distribution. According to the Almers theory, the logarithmic sensitivity function L(E) is introduced as follows:

$$L\left(E\right)=\ln\left(\frac{G_{Na}\left(E\right)}{G_{Na}^{max}-G_{Na}\left(E\right)}\right).$$

The asymptote passing through the first points of the L(E) function allows for evaluating Z_eff_, which is linearly proportional to the tangent of the asymptote slope [41].

### 4.6. Organotypic Tissue Culture Method

The effects of ACTH and two short ACTH-related fragments, Ac-KKRR-NH_2_ and H-PKKRRP-OH, on the neurite growth of DRG explants obtained from 10–12-day-old White Leghorn chicken embryos were studied in accordance with the protocol described in detail earlier [26,54]. The explants, control and experimental, were placed in sterile 40 mm collagen-coated Petri dishes and cultured in a medium consisting of Dulbecco’s modified Eagle’s medium with a low glucose content (1 g/L), 10% fetal bovine serum, and gentamicin (100 U/mL). The culturing medium for experimental explants also contained the studied peptide substances. The incubation of DRG explants was carried out at 37 °C and 5% CO_2_ in a CO_2_ incubator for three days. The explants were visualized using an Axio Observer Z1 microscope (Carl Zeiss, Oberkochen, Germany) and analyzed using ImageJ version 1.54g (National Institutes of Health, Bethesda, MD, USA) and ZEN 2012 (Carl Zeiss, Oberkochen, Germany) software. Morphometric evaluation of the neurite growth was carried out using the area index (AI), the parameter calculated as the ratio of the peripheral growth zone area to the central zone area. The average AI value of the control explants was taken as 100% [26,54].

The experiments were conducted using the equipment of the Confocal Microscopy Collective Use Center at the Pavlov Institute of Physiology of the Russian Academy of Sciences.

### 4.7. Conformational Analysis

Conformational analysis of the Ac-KKRR-NH_2_ and H-PKKRRP-OH molecules was carried out using TINKER 8.0 [55] and the MMFF94 force field [56]. An implicit account of the peptide solvation was performed in the framework of the GB/SA approach [57]. Two dielectric constant ε values were considered: ε = 10, which approximates the Na_V_1.8 channel binding site dielectric properties, and ε = 80, for aqueous solution. Around 100,000 single conformations were obtained for each of the two peptides applying the low-mode conformational search (LMOD) algorithm [58]. The lysine side chain amino groups and the arginine side chain guanidinium groups were positively charged.

To evaluate μ_ij_, the average distances between the positively charged side chain functional groups in the low-energy conformational space of the studied peptides, statistical data processing was performed using our custom C++ script. Firstly, the distances between the functional groups were calculated for every single peptide conformation. The distance between two positively charged side chain functional groups was considered as the distance between their centers of charge. For a lysine residue, it is the amino group nitrogen atom. For an arginine residue, it is the central carbon atom of the guanidinium group. Secondly, the distances between the functional groups were averaged over the entire ensemble of ~100,000 conformations, and over five low-energy subensembles containing all conformations with energies under a certain cutoff value relative to the global minimum. The chosen cutoff values were 3, 4, 5, 6, and 7 kcal/mol. The data obtained are presented as the mean ± standard error of the mean (SEM). Thirdly, the μ_ij_ values required to select the peptide conformations for further docking with the Na_V_1.8 channel were identified as the average distances between the positively charged side chain functional groups in a subensemble that contained between 1000 and 2000 of the generated conformations.

### 4.8. Peptide Docking with the Na_V_1.8 Channel Molecule

Peptide docking was performed with the human Na_V_1.8 channel molecule (PDB code 7WE4) [31]. At the first step, the target protein molecule was prepared. After the bound A-803467 ligand had been deleted from the PDB structure, hydrogens were automatically added to the heavy atoms using Avogadro 1.2. The arginine side chain guanidinium groups and lysine side chain amino groups were considered protonated, while the glutamic acid and aspartic acid side chain carboxylate groups were considered deprotonated. The entire Na_V_1.8 channel molecule was further energy minimized in the UFF force field [59] using OpenBabel 2.4. Energy minimization was carried out using the steepest descent protocol with the program default convergence criteria.

At the second step, the selection of the peptide conformations for further docking was carried out. All Ac-KKRR-NH_2_ and H-PKKRRP-OH conformations with energies below 7 kcal/mol relative to the global minimum and RMSD ˂ 4.0 were selected.

$$RMSD=\sum_{all\;cationic\;functional\;groups\;pairwise\;combinations\;i,j}\sqrt{\left|{\left(r_{ij}\right)}^{2}-{\left(\mu_{ij}\right)}^{2}\right|}$$

In the above expression, the summation is carried out over six pairwise combinations (i = 1, j = 2; i = 1, j = 3; i = 1, j = 4; i = 2, j = 3; i = 2, j = 4; i = 3, j = 4) of cationic side chain functional groups, where r_ij_ is the distance between the corresponding functional groups in a given peptide conformation, and μ_ij_ is the average distance between the same functional groups obtained in the conformational analysis.

At the third step, the selected peptide conformations were blindly docked four times each with the entire Na_V_1.8 channel molecule in AutoDock Vina 1.2.6 (Scripps Research, San Diego, CA, USA) [60] and further analyzed with AutoDockTools 1.5.6 (Scripps Research, San Diego, CA, USA) [61]. The protein was fit into a cubic box of 100 × 100 × 100 Å centered at the arithmetic mean of all Na_V_1.8 channel atomic coordinates. The docking grid was assigned automatically (0.375 Å in every dimension). The obtained docking poses were clustered manually, and the lowest-energy docking poses in each cluster were regarded as final. The exhaustiveness was set at 16. Ionic and hydrogen bonds were detected manually. The distance between the heavy atoms in a bond did not exceed 4.5 Å.

### 4.9. The Formalin Test

In the formalin test, the behavioral nociceptive responses induced by formalin, a chemical inflammatory stimulant, were recorded [62,63]. The response to formalin injection is characterized by two phases of nociceptive behavior. The first (acute, 3–5 min) phase is associated with the activation of peripheral nociceptors, primarily C-fibers. The second (tonic, 30–40 min) phase results from the inflammatory response mediated by histamine, serotonin, prostaglandins, and bradykinin [63,64].

In the first set of experiments, 7 experimental and 7 control adult male Wistar rats were used (average body weight 230 g). Five minutes prior to formalin injection, the experimental animals received an intraperitoneal injection of Ac-KKRR-NH_2_ (10.0 mg/kg in 1 mL Hanks’ solution), while the control animals received 1 mL of Hanks’ solution. In the second set, similarly, 7 experimental and 7 control rats (average body weight 250 g) were used, and H-PKKRRP-OH was administered intraperitoneally at 10.0 mg/kg. Finally, in the third set, 7 experimental and 8 control rats (average body weight 260 g) were used, and ACTH(1–24) was administered intraperitoneally at 2.5 mg/kg. ACTH(1–24) was applied as a fully functional shortened ACTH-based fragment retaining the N-terminal region of the hormone that includes the cationic KKRR motif of interest.

Formalin (2.5%, 50 µL) was injected subcutaneously into the plantar surface of the left hind paw, since the hind limbs are less often involved in the natural grooming of rats, which allows for more accurate registration of nociceptive patterns. The following nociceptive behaviors were recorded over a 60 min period after the formalin injection: the number of flexes and shakes of the affected limb (spinal level) and the duration of paw licking (supraspinal level).

### 4.10. Statistical Analysis

Statistical analysis was performed using STATISTICA 10.0 (StatSoft, Inc., Tulsa, OK, USA). The Kolmogorov–Smirnov test was used to assess the normality of data distribution. For the datasets with normal distribution (patch-clamp recordings and organotypic tissue culture experiments), comparisons were performed using Student’s *t*-test. Differences were considered statistically significant at *p* < 0.05. Data are presented as the mean ± standard error of the mean (SEM).

For the formalin test, statistical analysis was performed using Student’s *t*-test for comparisons between experimental and control groups within each phase (acute and tonic). In addition, a mixed-design ANOVA was applied to evaluate the effects of “phase” (acute and tonic) and “treatment” (control, Ac-KKRR-NH_2_, H-PKKRRP-OH, and ACTH(1–24)), followed by analysis of simple effects. Analyses were performed separately for flexing/shaking and licking behaviors. Differences were considered statistically significant at *p* < 0.05.

The methodological quality of the formalin test was assessed in accordance with ARRIVE guidelines, including randomization, blinding, and predefined exclusion criteria. Animals were randomly assigned to experimental groups, and blinding was maintained throughout the study. No animals or data points were excluded from the analysis.

## Figures and Tables

**Table 1:** The distances between the positively charged side chain functional groups in the Ac-KKRR-NH_2_ and H-PKKRRP-OH molecules, averaged over the entire ensemble of conformations (N_conf_ ≈ 100,000) and over several low-energy subensembles determined by the energy cutoff relative to the global minimum.

Cutoff, kcal/mol	Ac-KKRR-NH_2_			H-PKKRRP-OH		
	N_conf_	Distances, Å		N_conf_	Distances, Å	
None	102,192	K^1^–K^2^ 10.8 ± 2.3	K^2^–R^3^ 11.4 ± 2.4	101,668	K^1^–K^2^ 11.4 ± 2.0	K^2^–R^3^ 11.4 ± 2.2
		K^1^–R^3^ 12.5 ± 3.1	K^2^–R^4^ 13.0 ± 3.3		K^1^–R^3^ 10.4 ± 3.4	K^2^–R^4^ 10.7 ± 3.4
		K^1^–R^4^ 13.1 ± 4.2	R^3^–R^4^ 10.7 ± 2.5		K^1^–R^4^ 11.4 ± 3.8	R^3^–R^4^ 10.0 ± 2.7
7	7086	K^1^–K^2^ 10.8 ± 2.1	K^2^–R^3^ 10.9 ± 2.2	3704	K^1^–K^2^ 12.2 ± 1.9	K^2^–R^3^ 10.9 ± 2.3
		K^1^–R^3^ 12.5 ± 3.1	K^2^–R^4^ 11.8 ± 2.3		K^1^–R^3^ 10.8 ± 3.3	K^2^–R^4^ 10.9 ± 3.2
		K^1^–R^4^ 9.5 ± 3.2	R^3^–R^4^ 9.6 ± 2.7		K^1^–R^4^ 10.5 ± 4.2	R^3^–R^4^ 10.6 ± 2.4
6	3733	K^1^–K^2^ 10.8 ± 2.0	K^2^–R^3^ 10.8 ± 2.2	1756	K^1^–K^2^ 12.3 ± 1.9	K^2^–R^3^ 10.6 ± 2.4
		K^1^–R^3^ 12.4 ± 2.7	K^2^–R^4^ 11.6 ± 2.0		K^1^–R^3^ 10.9 ± 3.3	K^2^–R^4^ 10.9 ± 3.3
		K^1^–R^4^ 9.0 ± 2.9	R^3^–R^4^ 9.4 ± 2.7		K^1^–R^4^ 10.1 ± 4.3	R^3^–R^4^ 10.8 ± 2.3
5	1661	K^1^–K^2^ 10.8 ± 2.0	K^2^–R^3^ 10.8 ± 2.1	731	K^1^–K^2^ 12.5 ± 1.8	K^2^–R^3^ 10.4 ± 2.3
		K^1^–R^3^ 12.3 ± 2.6	K^2^–R^4^ 11.4 ± 1.9		K^1^–R^3^ 11.1 ± 3.3	K^2^–R^4^ 10.9 ± 3.2
		K^1^–R^4^ 8.5 ± 2.4	R^3^–R^4^ 9.3 ± 2.6		K^1^–R^4^ 9.7 ± 4.4	R^3^–R^4^ 10.8 ± 2.3
4	513	K^1^–K^2^ 10.8 ± 1.9	K^2^–R^3^ 10.9 ± 2.0	273	K^1^–K^2^ 12.9 ± 1.7	K^2^–R^3^ 10.0 ± 2.3
		K^1^–R^3^ 12.0 ± 2.3	K^2^–R^4^ 11.1 ± 1.7		K^1^–R^3^ 11.4 ± 3.2	K^2^–R^4^ 11.4 ± 3.1
		K^1^–R^4^ 8.0 ± 2.1	R^3^–R^4^ 9.0 ± 2.6		K^1^–R^4^ 9.0 ± 4.4	R^3^–R^4^ 10.8 ± 2.2
3	138	K^1^–K^2^ 11.0 ± 2.0	K^2^–R^3^ 11.1 ± 2.0	76	K^1^–K^2^ 13.5 ± 1.6	K^2^–R^3^ 9.4 ± 2.4
		K^1^–R^3^ 11.3 ± 2.0	K^2^–R^4^ 10.5 ± 1.5		K^1^–R^3^ 12.2 ± 3.0	K^2^–R^4^ 11.7 ± 3.3
		K^1^–R^4^ 7.4 ± 1.8	R^3^–R^4^ 9.0 ± 2.3		K^1^–R^4^ 8.4 ± 4.7	R^3^–R^4^ 10.7 ± 2.3

**Table 2:** Parameters of Ac-KKRR-NH_2_ and H-PKKRRP-OH conformational ensembles docked at the identified binding sites (A–F) in the Na_V_1.8 channel molecule: number of docked conformations (N_dock_), average docking scores (E_av_), lowest docking scores (E_min_), and highest docking scores (E_max_).

Binding Site	Ac-KKRR-NH_2_				H-PKKRRP-OH			
	N_dock_	E_av_, kcal/mol	E_min_, kcal/mol	E_max_, kcal/mol	N_dock_	E_av_, kcal/mol	E_min_, kcal/mol	E_max_, kcal/mol
A	124	−7.1 ± 0.3	−7.9	−6.3	89	−7.6 ± 0.4	−8.4	−6.6
B	121	−6.9 ± 0.3	−7.7	−6.1	85	−7.4 ± 0.4	−8.0	−6.5
C	6	−6.5 ± 0.2	−6.8	−6.1				
D	13	−7.1 ± 0.3	−7.6	−6.3	3	−7.5 ± 0.3	−7.7	−7.1
E	4	−6.9 ± 0.2	−7.1	−6.6	4	−7.7 ± 0.5	−8.3	−7.0
F	32	−7.0 ± 0.3	−7.6	−6.6	23	−7.6 ± 0.3	−8.2	−7.0

**Table 3:** Sequence alignment of human (hNa_V_1.8) and rat (rNa_V_1.8) Na_V_1.8 channel structures.

Binding Site	Segment	Isoform	Sequence *
D	S1_I_ + S2_I_	hNa_V_1.8	126 VSVHSWFS**L**FIT**V**TILV**N**CVCMTRT**D**LPEK**IE**YVF**T**VIY**T**FEALIKILA 174
		rNa_V_1.8	126 VSVHSWFS**I**FIT**I**TILV**N**CVCMTRT**D**LPEK**VE**YVF**T**VIY**T**FEALIKILA 174
	S3_I_ + S4_I_	hNa_V_1.8	188 PWNWLDFSVITLAYVG**T**AIDLRGISGL**R**TF**R**VL**R**AL**K**TVSVIPGL 232
		rNa_V_1.8	188 PWNWLDFSVITLAYVG**A**AIDLRGISGL**R**TF**R**VL**R**AL**K**TVSVIPGL 232
	S5_II_	hNa_V_1.8	790 LT**I**ILAIIVF**V**FALVGK**Q**LL**GE** 811
		rNa_V_1.8	789 LT**F**ILAIIVF**I**FALVGK**Q**LL**SE** 810
E	S1_II_ + S2_II_	hNa_V_1.8	660 **G**LVTDPFAELTITLCIVVNT**I**FMAM**E**H**HG**M**SPT**F**E**AMLQ**I**GNIVFT**I**FFT**A**EM**FV**KIIAF 719
		rNa_V_1.8	659 **E**LVTDPFAELTITLCIVVNT**V**FMAM**E**H**YP**M**TDA**F**D**AMLQ**A**GNIVFT**V**FFT**M**EM**AF**KIIAF 718
	S3_II_ + S4_II_	hNa_V_1.8	728 KWNIFDC**I**IVTVSLL**E**L**GVA**KKG**S**L**S**VL**RS**F**R**LL**R**VF**K**LA**K**SWPTL 773
		rNa_V_1.8	727 KWNIFDC**V**IVTVSLL**E**L**SAS**KKG**S**L**S**VL**RT**F**R**LL**R**VF**K**LA**K**SWPTL 772
	S5_III_	hNa_V_1.8	1284 VLLVCLIFWLIFSIMGV**N**LFAG**K**F**WR**C**I** 1311
		rNa_V_1.8	1285 VLLVCLIFWLIFSIMGV**N**LFAG**K**F**SK**C**V** 1312
F	S1_III_ + S2_III_	hNa_V_1.8	1148 IVEHSWFESFIIFMILLSSG**S**LAF**E**D**Y**YL**DQ**KP**T**VK**AL**L**E**YT**D**RVFTFIFVFEMLLKWVAYGF 1210
		rNa_V_1.8	1149 IVEHSWFESFIIFMILLSSG**A**LAF**E**D**N**YL**EE**KP**R**VK**SV**L**E**YT**D**RVFTFIFVFEMLLKWVAYGF 1211
	S3_III_ + S4_III_	hNa_V_1.8	1217 AWCWLDFLIVNISL**I**SL**T**AKILEYS**E**VA**P**I**K**AL**R**TL**R**AL**R**PL**R**ALSRF 1264
		rNa_V_1.8	1218 AWCWLDFLIVNISL**T**SL**I**AKILEYS**D**VA**S**I**K**AL**R**TL**R**AL**R**PL**R**ALSRF 1265
	S5_IV_	hNa_V_1.8	1610 IGLLLFLVMFIYSIFGM**SS**F**RH**V 1632
		rNa_V_1.8	1611 IGLLLFLVMFIYSIFGM**AS**F**AN**V 1633
	ECL before S6_IV_	hNa_V_1.8	1671 LNTGPPYCDPNLPNSN**GT**R**GDC**GSPAV**G** 1698
		rNa_V_1.8	1672 LNTGPPYCDPNLPNSN**GS**R**GNC**GSPAV**I** 1699

* The amino acid residues of the Na_V_1.8 channel molecule mentioned throughout the manuscript (the residues involved in intermolecular ionic and hydrogen bonds with the studied short ACTH-related cationic peptides, the residues involved in intramolecular ionic and hydrogen bonds with the S4 voltage sensor residues, and the S4 voltage sensor residues per se) are highlighted with bold red characters. The unconserved residues within the aligned sequences are highlighted with bold, underlined characters.

## Data Availability

The original contributions presented in this study are included in the article. Further inquiries can be directed to the corresponding author.

## Abbreviations

The following abbreviations are used in this manuscript:

ACTH	Adrenocorticotropic hormone
HPA axis	Hypothalamic–pituitary–adrenal axis
PD	Pore domain
ECL	Extracellular loop
DRG	Dorsal root ganglia
AI	Area index
